# *Vitellogenin-like A*–associated shifts in social cue responsiveness regulate behavioral task specialization in an ant

**DOI:** 10.1371/journal.pbio.2005747

**Published:** 2018-06-06

**Authors:** Philip Kohlmeier, Barbara Feldmeyer, Susanne Foitzik

**Affiliations:** 1 Institute of Organismic and Molecular and Evolution, Johannes Gutenberg University Mainz, Mainz, Germany; 2 Senckenberg Biodiversity and Climate Research Centre, Senckenberg Gesellschaft für Naturforschung, Frankfurt am Main, Germany; The Rockefeller University, United States of America

## Abstract

Division of labor and task specialization explain the success of human and insect societies. Social insect colonies are characterized by division of labor, with workers specializing in brood care early and foraging later in life. Theory posits that this task switching requires shifts in responsiveness to task-related cues, yet experimental evidence is weak. Here, we show that a *Vitellogenin* (*Vg*) ortholog identified in an RNAseq study on the ant *T*. *longispinosus* is involved in this process: using phylogenetic analyses of *Vg* and *Vg-like* genes, we firstly show that this candidate gene does not cluster with the intensively studied honey bee *Vg* but falls into a separate *Vg-like A* cluster. Secondly, an experimental knockdown of *Vg-like A* in the fat body caused a reduction in brood care and an increase in nestmate care in young ant workers. Nestmate care is normally exhibited by older workers. We demonstrate experimentally that this task switch is at least partly based on *Vg-like A*–associated shifts in responsiveness from brood to worker cues. We thus reveal a novel mechanism leading to early behavioral maturation via changes in social cue responsiveness mediated by *Vg-like A* and associated pathways, which proximately play a role in regulating division of labor.

## Author summary

In social insects such as ants and bees, workers specialize in different tasks. This specialization is thought to be regulated via response thresholds to task-specific cues, which vary between workers conducting different tasks. Whether a worker takes care of the brood, cares for other workers, or leaves the nest to search for food is influenced by age, fat content, and the expression of associated genes. In the ant *Temnothorax longispinosus*, workers specializing in brood care are younger and exhibit a high expression of the gene *Vg-like A*. Here, we demonstrate that young workers reduce brood care activity upon down-regulation of *Vg-like A*. Simultaneously, they increase care for adult nestmates, a behavior typically exhibited by older workers. We show experimentally that *Vg-like A* down-regulation alters perception of social cues: a shift in the responsiveness from chemical cues from brood to adult worker underlies the behavioral switch to nestmate care. Hence, the expression of *Vg-like A* and its associated pathways influences task choice in ants and is involved in the regulation of division of labor. Copies of *Vg-like A* are present in other ants and further social and solitary insects, and future studies will reveal the role of this gene in these organisms.

## Introduction

Division of labor—the distribution of work among group members—is a characteristic trait of many social species, including humans, other social mammals, and social insects. An important component of division of labor is task specialization, which increases the efficiency in performing a task and hence contributes to the evolutionary success of division of labor [1–4]. The occurrence and benefits of task specialization, however, might be context specific and are occasionally questioned [5–11]. The assignment of workers to their respective roles is rarely regulated by the queen or other dominant group members and is only in a few cases genetically determined [12]. Instead, division of labor is typically self-organized, and specialization of workers in specific tasks is affected by a variety of factors, including morphology, age, fecundity, and corpulence. For instance, in many ant and bee species, hard-wired developmentally regulated physiological and/or morphological castes can be found, resulting in highly specialized workers, e.g., ant soldiers [2,13]. In species with a monomorphic worker caste, task specialization is often not a lifetime choice but typically undergoes multiple changes over a worker’s life: recently hatched, corpulent, and fertile workers stay on the brood pile and tend the brood. Later in life, following a shrinkage of lipid reserves and ovaries, workers switch to extranidal tasks such as foraging [2,14–20], suggesting that the behavioral progression from internal to external tasks is rigid and unidirectional.

However, workers in many social insect taxa retain a certain behavioral flexibility. For example, in primitively eusocial species, such as some wasps and bumble bees, worker task choice is only weakly linked to age [21–27] (but see [28,29]), and workers can frequently switch between inside tasks and foraging [28]. In ants and honey bees, foragers can switch back to inside tasks in case of a sudden increase in brood-care demands [30–35]. In the reverse case—i.e., a removal of foragers—young workers accelerate their behavioral progression from inside to outside tasks (e.g., [32,35], but see, e.g., [36,37]). Such high behavioral flexibility is inconsistent with the concept of behavior being directly linked to age and physiological status, as these factors are impossible (in the case of age) or difficult (in the case of physiological status [38]) to adapt.

Instead, theoretical considerations based on mathematical models led to the development of two prominent models explaining task choice and division of labor in social insects. The foraging for work (FFW) model states that division of labor is generated via the spatial distribution of tasks in more or less distinct areas of the nest. As workers differ in their typical spatial position and seek open jobs in their vicinity, workers take over different duties [39–41]. The observed age-dependent choice of tasks can thus be explained by young workers hatching from the centrally located brood pile and physically transposing older workers to the periphery, where they then encounter different tasks [42]. The response threshold (RT) model is based on the idea that task choice is controlled by a set of internal RTs regulating the responsiveness of a worker toward different task-related stimuli [43,44]. To induce a behavioral response in a worker, the task-related stimulus has to exceed the internal RT. These thresholds are influenced by gene networks and cascades, which, in turn, are affected by the aforementioned physiological parameters such as age. In line with that, brood carers and foragers exhibit broad differences in gene expression and splicing [32–34,45–50]. Given that the expression of behaviorally linked genes is influenced by physiology—determined by, e.g., age, fertility, and corpulence—typical patterns of task allocation can be explained by the RT model [44].

Unlike a solely physiology-based behavioral progression of workers, both the FFW and the RT model can explain typical patterns of age polyethism while simultaneously allowing for behavioral flexibility: if the demand for a certain task—e.g., brood care—increases, both the likelihood to encounter such open jobs (FFW model) and the stimulus intensity associated with this demand (RT model) will rise, and even workers with high thresholds for brood care–related tasks, such as foragers, will switch to take over this job.

Recent studies on honey bees provide experimental evidence for the importance of this stimulus–RT–gene expression association in influencing task choice. In particular, forager bees exhibit a high expression of the *Amfor* gene [51], which increases their responsiveness toward sucrose solution and results in elevated nectar-foraging activity [52]. The role of *foraging* and associated pathways in division of labor in other social Hymenoptera, however, appears to be less clear [53–56].

In contrast to foraging, brood care activity is likely influenced by the insulin-like growth factor 1 (IGF-1) pathway, an important endocrine network associated with fertility and behavior in insects [57–59]. Well-characterized members of this network are *vitellogenin* (*Vg*) genes and the associated juvenile hormone (JH): in honey bees, high expression of the single *Vg* copy is linked positively to brood care behavior and negatively to the onset of foraging [60–62]. When *Vg* is down-regulated, JH titers increase and result in reduced brood care, precocious foraging, and a forager-like gene expression profile [60–64]. Although the link between *Vg* and brood care behavior is well documented in bees and was even expanded to noneusocial systems [65], little is known about how changes in the expression of *Vg* and downstream effects contribute to alterations in behavior and task choice. One suggested mechanism is that a down-regulation of *Vg* is involved in increasing gustatory responsiveness and might thereby translate into precocious foraging in honey bees [66]. Moreover, it is still unclear to what extent functional annotations of and conclusions based on the honey bee *Vg* can be expanded to other social Hymenoptera such as ants, bumble bees, and wasps. Firstly, in contrast to honey bees, the association between *Vg* and JH is less clear in other social insects, suggesting fundamental changes in the underlying networks regulating behavior, e.g., in some ants [67–69]. Secondly, *Vg* underwent several duplication events followed by diversification and subfunctionalization in ants and termites, potentially resulting in different *Vg* orthologs taking over different functions in physiology—namely, fertility and aging—and behavior, including division of labor [46,70–75]. Thirdly, 3 new clusters of *Vg-like* genes have recently been discovered in many social and solitary species [73]. Despite their structural similarity to the conventional *Vg*s, their function is unknown but has been suggested to be linked to inflammation and oxidative stress response [76]. Fourthly, a clear picture of *Vg* copy numbers, their phylogenetic relationships, and functions is lacking, and nomenclature of the different *Vgs* in different organisms is rather eclectic.

The identification of gene pathways and networks involved in the regulation of behavior requires controlling for confounding physiological factors to disentangle the effects of physiology and behavior on gene expression [32]. We thus experimentally manipulated the age structure of ant colonies by removing either all old or all young workers in a full factorial design regarding task (brood carer, forager), age (young, old), and fertility (fertile, infertile) [34]. This allowed us to investigate the independent contribution of each factor on behavior as well as the underlying gene expression patterns [34]. Among the genes in which expression was linked to behavior—in this case, brood care behavior—*Vg-like A* [73] stood out as a highly promising candidate to influence task choice and division of labor, as it exhibited the strongest expression difference between brood carers and foragers (false discovery rate [FDR] = 5.45 × 10^−18^), and its expression was independent from age (FDR = 0.99) and fertility (FDR = 0.99) [34].

Here, we use a series of RNA interference (RNAi)-induced *Vg-like A* knockdowns followed by behavioral assays, physiological and chemical measurements, and tissue-specific quantification to functionally characterize this gene in *T*. *longispinosus* and to elucidate its role in task choice and, by that, in division of labor. In particular, we investigated (i) how worker behavior is influenced by age and task demands, (ii) whether *Vg-like A* influences behavior and behavioral progression and how *Vg-like A* interacts with worker age, (iii) whether the *Vg-like A*–associated behavioral regulation is modulated via responsiveness to task-related stimuli (as predicted by theory), (iv) the tissue-specific expression of *Vg-like A* and other *Vg-like* and *Vg* orthologs and whether these are affected by *Vg-like A* knockdown, and (v) the phylogenetic position of *Vg-like A* relative to other *Vgs* and *Vg-like* genes within Hymenoptera, including both solitary and social insect taxa.

Answering these questions will contribute to our understanding of the mechanistic underpinnings of behavioral plasticity and how these mechanisms respond to and interact with a worker’s social environment. Furthermore, investigating the role of *Vg* orthologs and their associated pathways in the regulation of behavior in other systems than the honey bee offers the unique possibility to reconstruct the mechanistic underpinnings of different evolutionary routes toward eusociality.

## Results

### Age polyethism and behavioral flexibility

**Fig 1:**
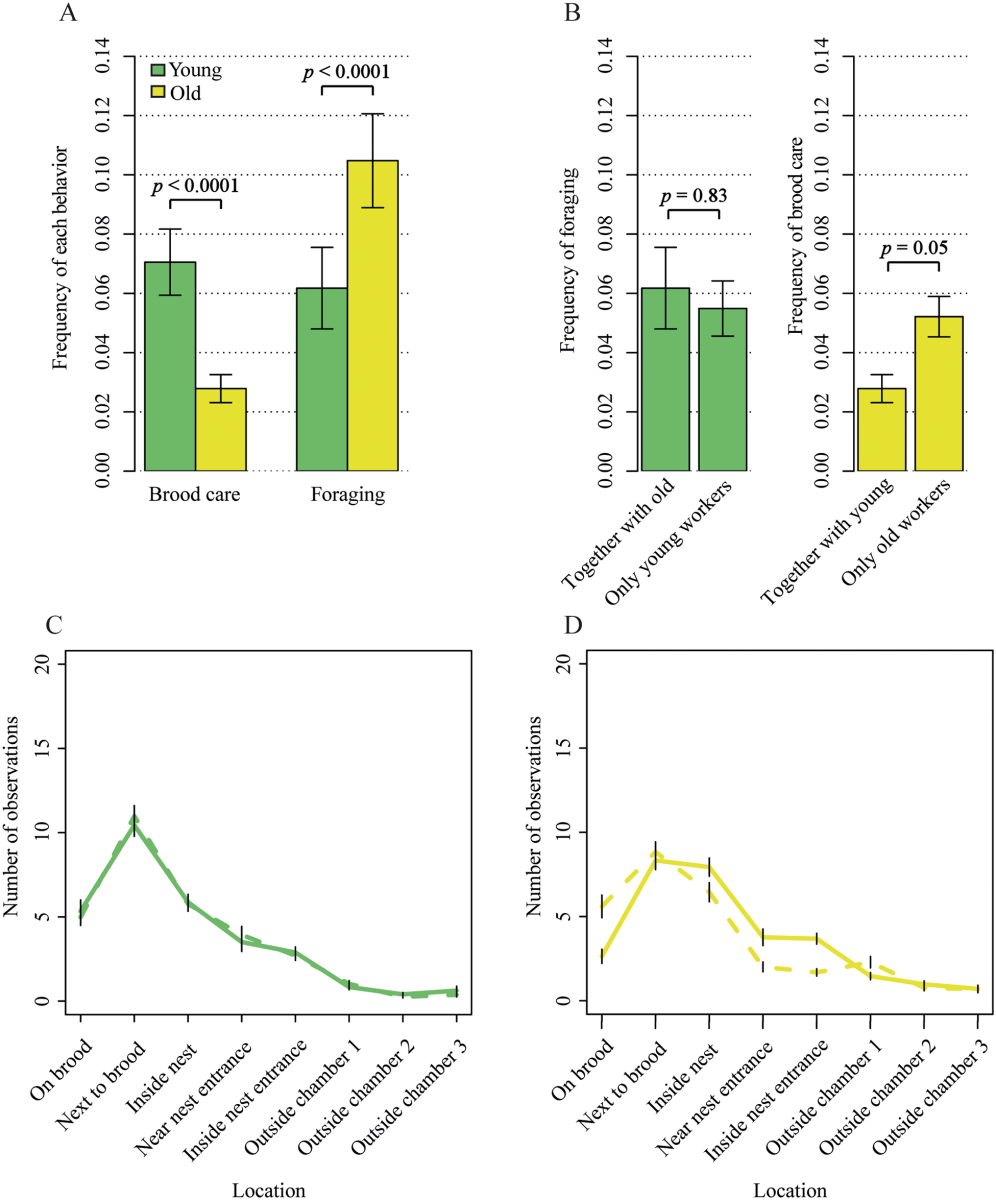
(A) In colonies with both age cohorts present (control), young workers preferably tended the brood, whereas old workers foraged. (B) Behavioral flexibility is age dependent. Young workers did not increase the frequency of foraging after old worker removal (left), whereas old workers increased brood-caring behavior following young worker removal. (C) Mean position of young workers did not depend on being together with old workers (solid line) or without old workers (dashed line). Vertical lines show standard error. (D) Old workers shifted their location towards brood pile in response to young worker removal (dashed line). Solid line shows positions of old workers when together with young workers.

**Fig 2:**
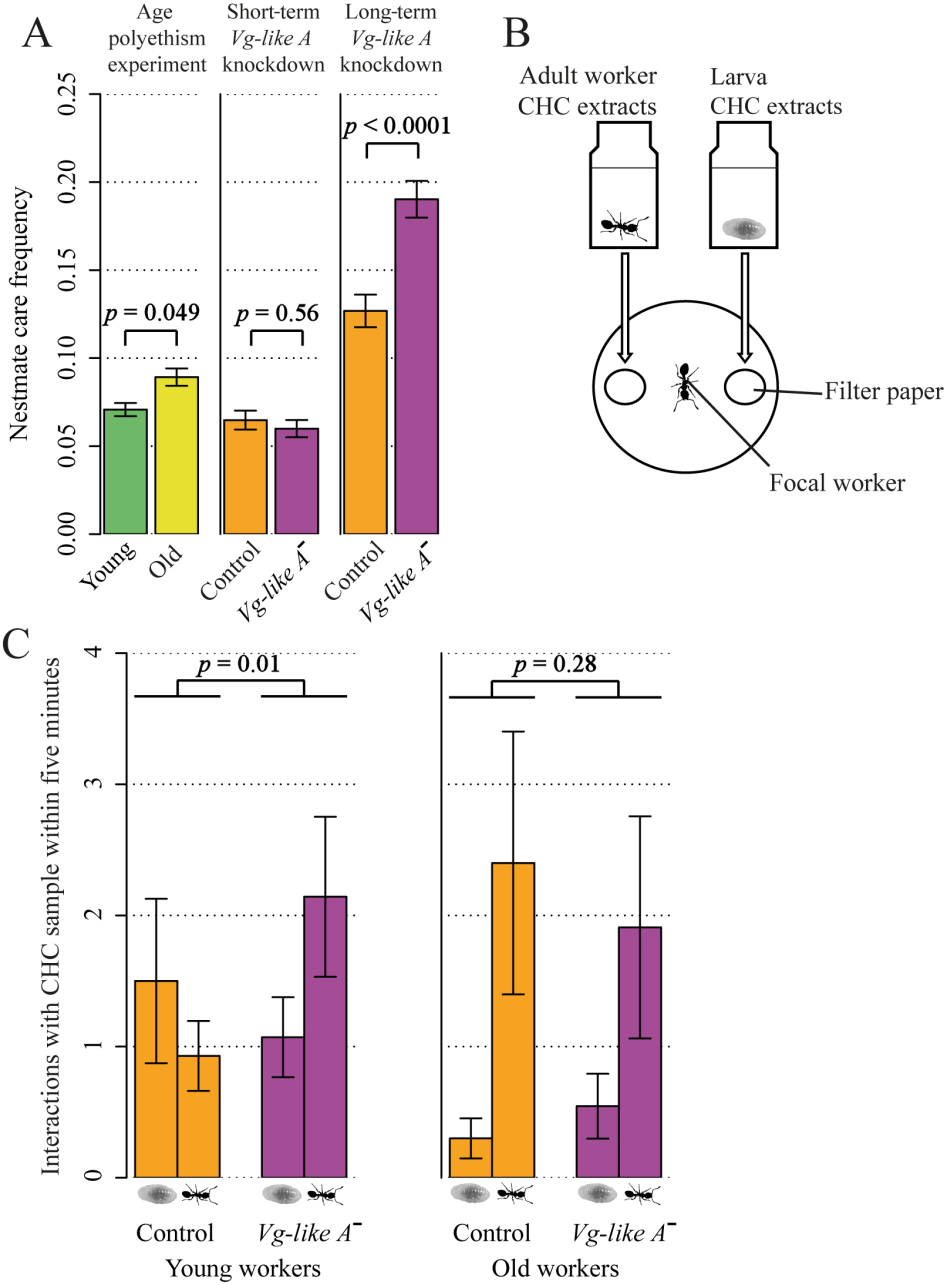
Long-term *Vg-like A* knockdown increases nestmate care behavior via alterations in cue responsiveness. (A) Under *Vg-like A* knockdown, young brood carers increased adult nestmate care, which we did not find under short-term knockdown. Nestmate care was usually taken over by the old workers. (B) Young (i.e., 3 months old) and old (i.e., 1 year or older) workers from control or *Vg-like A*^*−*^ treatment were tested for preference for worker and brood CHC extracts. (C) In young workers, *Vg-like A* knockdown resulted in a change in cue responsiveness for brood and worker CHCs, whereas the knockdown did not affect old workers. In the control, young and old workers differed in the number of interactions with each extract (Binomial GLMM: *p* = 0.0003), whereas this was not the case under *Vg-like A* knockdown (Binomial GLMM: *p* = 0.32). CHC, cuticular hydrocarbon; GLMM, generalized linear mixed model; Vg, vitellogenin.

We manipulated colony demography in field-collected colonies of the ant *T*. *longispinosus* by removing either (i) all newly hatched (termed “young”) workers in the lab, (ii) all workers at least 1 year old (“old”), or (iii) half of each age cohort as a control. Based on subsequent behavioral observations of 12 workers per nest, we detected the typical age-dependent division of labor in our control colonies consisting of both young and old workers. In particular, the “frequency of brood care” was influenced by an interaction between “age” and “colony treatment” (generalized linear mixed model [GLMM]: family = quasi-Poisson, χ^2^ = 3.8, *p* = 0.049; Fig 1A), and brood care was mainly conducted by young workers in control colonies (Model estimate: z = 4.5, *p* < 0.0001). The “frequency of foraging,” in contrast, was independent from this interaction (GLMM: family = quasi-Poisson, χ^2^ = 0.7, *p* = 0.419; Fig 1B) and only affected by “age” (GLMM: χ^2^ = 15.6, *p* < 0.0001), with old workers foraging more often than young workers irrespective of “colony treatment” (GLMM: χ^2^ = 0.1, *p* = 0.742). When removing all young workers from a colony, old workers responded with elevating their “frequency of brood care” to a level close to significance (Model estimate: z = 2.0, *p* = 0.051) and up to the level of young workers (Model estimate: z = 0.7, *p* = 0.509). In the reverse case, under old worker removal, young workers did not increase their “frequency of foraging” (Model estimate: *t* = 0.2, *p* = 0.831). These age-dependent differences in plasticity were mirrored in the spatial location of workers, which were influenced by an interaction between “age” and “treatment” (permutational multivariate ANOVA [PERMANOVA]: F = 4.0, *p* = 0.008). In particular, old workers moved to the inside of the nest when all young workers were removed (PERMANOVA: F = 8.0, *p* = 0.0001), whereas young workers did not relocate to the nest periphery in the reverse case (PERMANOVA: F = 0.1, *p* = 0.959). In honey bees, the care of adult nestmates has been described as an intermediate step in the behavioral progression from brood care to foraging [77]. In line with this, we found adult nestmate care—i.e., feeding, grooming, or carrying of adult workers—to be preferably taken over by old workers (GLMM: χ^2^ = 3.8, *p* = 0.049; Fig 2A).

### *Vg-like A* regulation of behavior and behavioral progression

We conducted a series of knockdown experiments to functionally annotate *Vg-like A* and to reveal the impact of the pathway and network involving *Vg-like A* on task choice, behavioral progression, and division of labor. We first conducted a short-term (i.e., 7-day) Dicer-substrate small interfering RNA (dsiRNA)-mediated whole-colony knockdown of *Vg-like A* by providing dsiRNA fragments dissolved in sucrose solution for oral uptake. This resulted in a 62.9% down-regulation of *Vg-like A* in the fat body of brood carers (see below).

**Fig 3:**
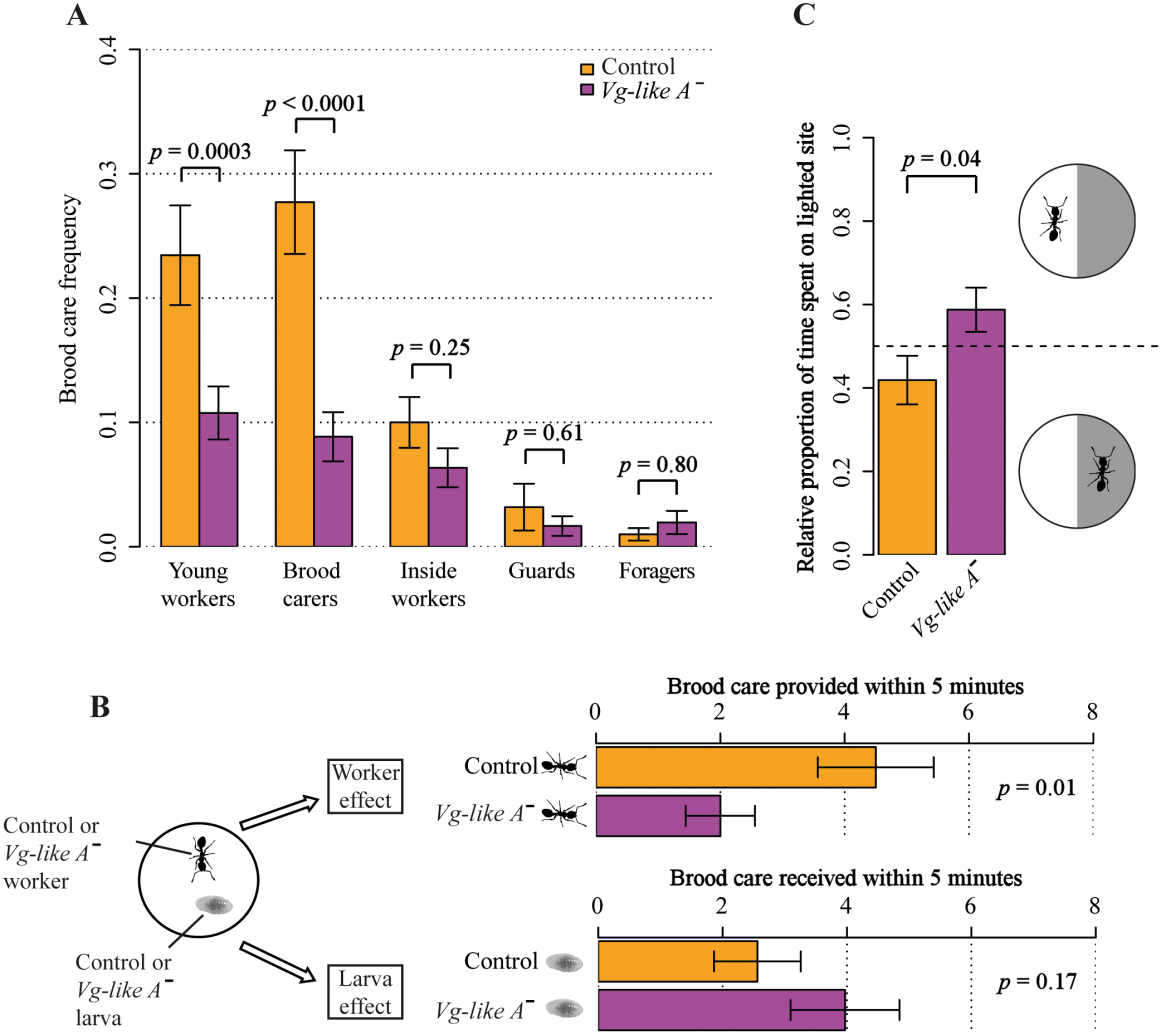
Short-term knockdown of *Vg-like A* reduced brood care behavior. (A) Brood care frequency was influenced by an interaction between treatment (control, *Vg-like A*^*−*^) and caste (GLMM: *p* < 0.0001). A down-regulation of *Vg-like A* decreased brood care behavior in young workers and brood carers but not in other castes. (B) Workers and larvae were tested (*n* = 65) in a full-factorial design regarding worker and larval treatment (control, *Vg-like A*^*−*^) in individualized tests. Brood care conducted by workers was counted for 5 minutes every 15 seconds. *Vg-like A* knockdown–associated decrease in brood care can be explained by worker treatment but not by larval treatment. (C) *Vg-like A*^−^ brood carers exhibited lower light aversion. Brood carers of both treatments were individually transferred to half-darkened petri dishes. For 60 minutes, position (darkened or lighted site of the dish) was recorded every 5 minutes. Dashed line at 50%, i.e., same time spent on each site. GLMM, generalized linear mixed model; *Vg*, vitellogenin.

**Fig 4:**
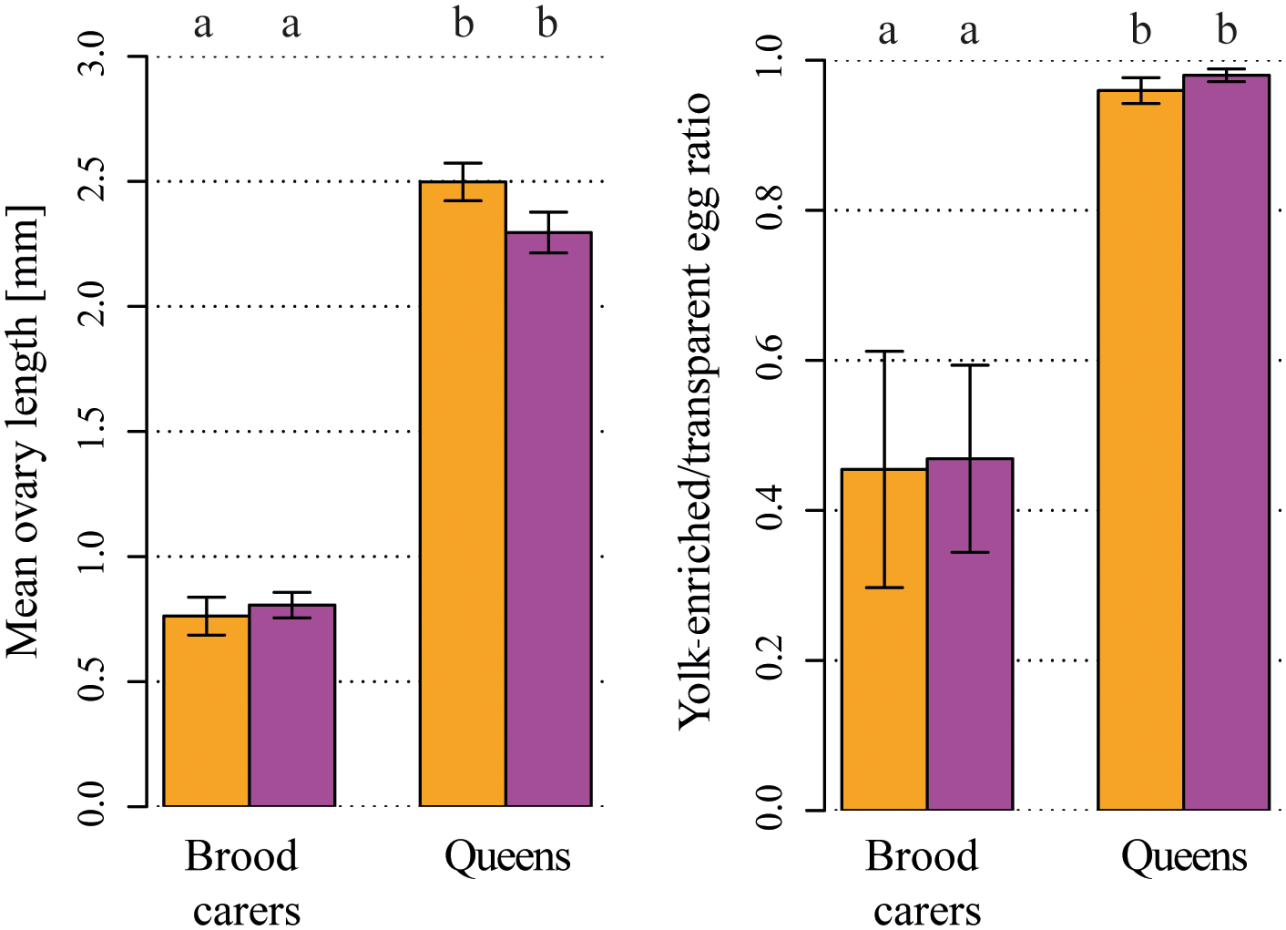
Fertility of brood carers and queens was not influenced by *Vg-like A* knockdown. Mean ovary length (left) and yolk-enriched/transparent egg ratio (right) exhibited strong caste differences but were independent of *Vg-like A* knockdown (Orange: control; purple: *Vg-like A* knockdown). *Vg*, vitellogenin.

*Vg-like A* down-regulation reduced brood care behavior in young (i.e., recently hatched) workers and field-collected brood carers but not in other behavioral castes, which generally conducted little brood care (Fig 3A). This reduction in brood care cannot be explained by an overall decrease in behavioral activity due to potentially harming effects of the *Vg-like A* knockdown, as the “frequency of inactivity” was independent from “treatment” (GLMM: χ^2^ = 1.3, *p* = 0.255; S1 Fig). As we provided dsiRNA for the entire colony, a reduction in brood care could potentially be explained by *Vg-like A* knockdown–induced alterations in larval rather than worker behavior. We tested this by performing individualized brood care tests with a full factorial design regarding worker and larva dsiRNA treatment. The amount of brood care provided within 5 minutes was only influenced by “worker treatment” (GLMM: χ^2^ = 5.5, *p* = 0.019; Fig 3B) but independent from “larval treatment” (GLMM: χ^2^ = 1.8, *p* = 0.176) or an interaction between both factors (GLMM: χ^2^ = 0.8, *p* = 0.361). Despite the reduction in brood care, we did not detect an effect of “treatment” on the “frequency of foraging” (GLMM: treatment: χ^2^ = 0.0, *p* = 0.935; caste: χ^2^ = 68.1, *p* < 0.0001; interaction: χ^2^ = 0.2, *p* = 0.997; S2 Fig). The only indication, though weak, for an ongoing transition toward outside tasks was a reduced light aversion of *Vg-like A*^*−*^ brood carers (*t* test: *t* = 2.1, *p* = 0.040, Fig 3C). As *Temnothorax* workers have a life expectancy of up to 7 years [78], much higher than the lifespan of honey bee workers [79], we tested whether a prolonged *Vg-like A* knockdown induces foraging in brood carers and repeated our experiment with two changes in the setup: Firstly, we increased the knockdown period from 7 to 33 days (named “long-term knockdown”). Secondly, as we were specifically interested in *Vg-like A* knockdown–associated accelerated behavioral maturation, we exclusively observed 3-month-old brood carers. Again, a reduction in brood care occurred (GLMM: χ^2^ = 31.1, *p* < 0.0001; S3 Fig), but still no increase in foraging activity (GLMM: χ^2^ = 0.1, *p* = 0.794; S4 Fig). Furthermore, fecundity—measured as the mean ovary length and the ratio of yolk-enriched/transparent eggs—was, as expected, higher in queens than in workers (GLMM: mean ovary length: χ^2^ = 215.7, *p* < 0.0001; yolk-enriched/transparent egg ratio: χ^2^ = 136.7, *p* < 0.0001; Fig 4) but independent from *Vg-like A* treatment (GLMM: mean ovary length: χ^2^ = 0.2, *p* = 0.639; yolk-enriched/transparent egg ratio: χ^2^ = 0.2, *p* = 0.677) or an interaction between “caste” and “treatment” (GLMM: mean ovary length: χ^2^ = 0.8, *p* = 0.370; yolk-enriched/transparent egg ratio: χ^2^ = 0.0, *p* = 0.967). A comparison of cuticular hydrocarbon (CHC) profiles of brood carers and foragers of both treatments revealed a strong caste effect (PERMANOVA: pseudo-F = 8.072, *p* = 0.001, S5 Fig)—i.e., the two behavioral castes differ in chemical profile—but no effect of *Vg-like A* knockdown (PERMANOVA: pseudo-F = 0.618, df = 1, *p* = 0.70) or an interaction between “caste” and “treatment” (Permanova: pseudo-F = 0.532, df = 1, *p* = 0.67) on cuticular chemistry. However, young *Vg-like A*^*−*^ brood carers increased adult nestmate care (Fig 2A), a task found before to be typically taken over by old workers. Thus, our results suggest that a down-regulation of *Vg-like A* accelerates behavioral progression from brood care to adult nestmate care but not all the way to foraging.

### *Vg-like A*–associated responsiveness to task-related stimuli

The most likely proximate mechanism underlying the observed behavioral progression is a shift in responsiveness from brood- to adult worker–related stimuli. To test this hypothesis, we compared the responsiveness of young (30-day-old) and old (1-year-old) workers to CHC extracts of larvae and adult workers under control and *Vg-like A* knockdown conditions (Fig 2B). In the control with unmanipulated *Vg-like A* expression, young workers and old workers differed in their preference (Binomial GLMM: z = 3.6, *p* = 0.0002; Fig 2C), but this difference vanished under *Vg-like A* knockdown (Binomial GLMM: z = 1.0, *p* = 0.318). In response to a *Vg-like A* down-regulation, young workers changed their preference to adult worker odors, thereby resembling old workers (Binomial GLMM: z = 2.5, *p* = 0.013), whereas old workers did not change their behavior (Binomial GLMM: z = 1.1, *p* = 0.282).

### Tissue-specific expression of *Vg* and *Vg-like* genes in *T*. *longispinosus*

**Fig 5:**
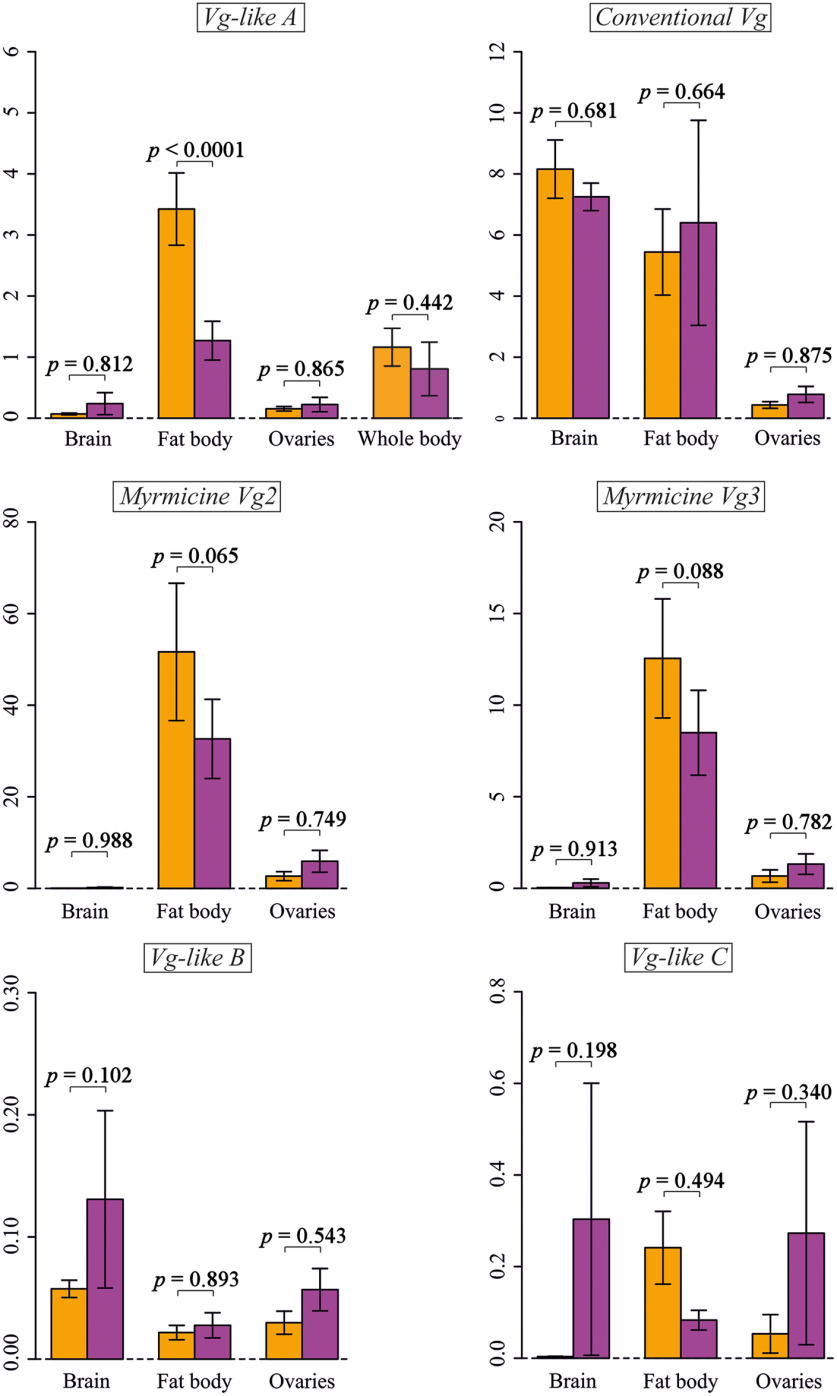
Tissue-specific expression of *Temnothorax Vg* and *Vg-like* genes. mRNA of each gene was quantified via qPCR relative to *alpha tubulin* expression. A knockdown of *Vg-like A* (purple bars) reduced *Vg-like A* expression in the fat body. The expression of no other *Vg* or *Vg-like* gene was altered by a *Vg-like A* knockdown. Orange bars: control. qPCR, quantitative real-time PCR; Vg, vitellogenin.

We quantified the expression of *Vg-like A* and all other *Vg* and *Vg-like* genes in the brain, fat body, and ovaries of *T*. *longispinosus* brood carers via quantitative real-time PCR (qPCR). We dissected workers and pooled the tissues of 5 workers from 22 colonies, resulting in 11 samples for each tissue for the RNAi treatment and control, respectively. *Vg-like A* expression was influenced by an interaction between “tissue” and “treatment” (GLMM: χ^2^ = 20.8, *p* < 0.0001). In particular, *Vg-like A* was down-regulated by 62.9% in the fat body (Model estimate: *t* = 5.3, *p* < 0.0001), the tissue in which it was most highly expressed (fat body versus brain, model estimate: *t* = 8.2, *p* < 0.0001; fat body versus ovaries, model estimate: *t* = 8.0, *p* < 0.0001), but not in the brain and in the ovaries (Fig 5). The down-regulation of *Vg-like A* was not apparent in the whole-body samples (Wilcoxon test: W = 40, *p* = 0.442). The expression of *CVg*, *MVg*2 and *MVg*3, and *Vg-like B* and *Vg-like C* was not altered by *Vg-like A* knockdown in either of the 3 tissues (all *p*-values > 0.05, Fig 5 for statistical details). However, each of these genes showed clearly tissue-specific expression, with, e.g., the myrmicine *Vgs* (*MVg*2 and *MVg*3) being mainly expressed in the fat body (*MVg*2: fat body versus brain, model estimate: *t* = 5.2, *p* < 0.0001; fat body versus ovaries, model estimate: *t* = 4.9, *p* < 0.0001; *MVg*3: fat body versus brain, model estimate: *t* = 5.4, *p* < 0.0001; fat body versus ovaries, model estimate: *t* = 5.1, *p* < 0.0001).

### Resolving the *Vg* and *Vg-like* phylogeny in ants and hymenopterans

**Fig 6:**
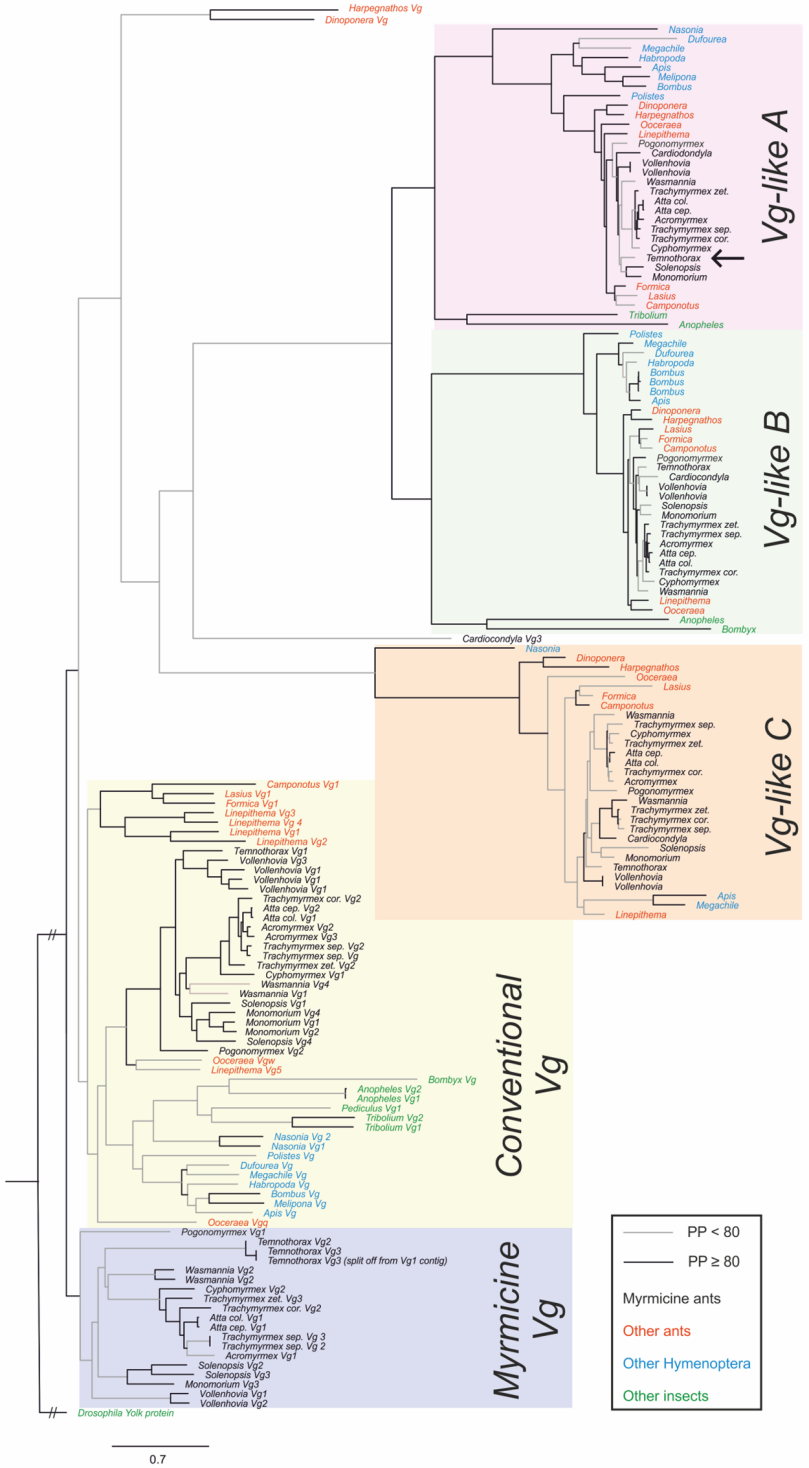
RAxML maximum likelihood tree of all *Vg* and *Vg-like* copies found in 33 social and solitary insects. One cluster of conventional *Vg*s, 3 *Vg-like* clusters, and 1 myrmicine specific cluster were identified. *Vg/Vg-like* copies are color coded. Within each cluster, genes are grouped according to taxonomic groups. The gene knocked down in this study fell into the *Vg-like A* cluster (indicated by the black arrow). PP, posterior probability; Vg, vitellogenin.

The BLAST searches of our *Vg* contigs resulted in highly ambiguous annotations, possibly due to the highly conserved structural domains [73]. To obtain reliable *Vg* cluster membership, we used *Vg* sequences of 2 publications [72,73] as queries for an extensive BLAST search of *Vg* copies in a total of 34 insect genomes. A number of *Vg* copies, as well as *Vg-likes*, have previously not been annotated in several taxa (complete list of *Vg* copies per species in S1 Table). Moreover, some genome annotations contained “fused” protein sequences of two *Vg*s combined into one (see S2 Table for details). We split these “fused” proteins into 2 protein sequences. We used our BLAST results and sequences from the published alignments to construct an extensive maximum likelihood tree with RaXml [80] containing 5 distinct *Vg* clusters (Fig 6). The *Vg-like* genes (according to [73]) form 3 distinct clusters, which are present in all hymenopterans, with some of them occurring in nonhymenopteran taxa such as mosquitos, butterflies, and beetles. *Vg-like C* was found in Hymenoptera only. The *Vg* genes form 2 distinct clusters, with *CVg* being present in all investigated insects, whereas the second cluster, *MVg*, occurs only in ants of the subfamily Myrmicinae. Within each gene cluster, the expected taxonomic groups are recovered, with the formicine, myrmicine, and ponerine species clustering together (Supporting information) as well as the apoid bees. The number of *CVgs*, *MVg*, and *Vg-likes* is highly variable between taxa, pointing to species-specific duplications and losses. The *T*. *longispinosus* contig of interest for this study grouped to the *Vg-like A* cluster and was annotated as such.

## Discussion

The likelihood that a social insect worker takes over an open task is influenced by a complex interaction of age, physiology, and gene expression influencing internal RTs for task-related stimuli. The expression of these genes typically follows age-dependent physiological changes but—under certain conditions—can be modified in response to increased demand for a certain task. *Vgs* are known for their role in fertility and division of labor in social insects [60–67,71]. Here, we show that in the ant *T*. *longispinosus*, typical patterns of age-dependent division of labor can be found, with young workers tending the brood and old workers taking over the care of adult nestmates and foraging. Old workers were more plastic in their behavior and could return to brood care tasks if necessary, whereas young workers failed to accelerate their behavioral progression toward foraging. Our detailed functional annotation of *Vg-like A*—a gene highly expressed in brood carers, independent of their age and fertility status [34]—showed that *Vg-like A*, or the networks it is embedded in, is involved in mediating cue responsiveness and may hence play a role in the regulation of division of labor. In particular, *Vg-like A* down-regulation caused young workers to decrease their investment into brood care and to switch to the care for adult nestmates, a behavior typically exhibited by workers older than 1 year. These nestmate care workers are probably functionally similar to middle-aged honey bee workers that quit their brood care duties [81] and exhibit a broad behavioral repertoire of various intranidal tasks, including nest building, nectar receiving, and guarding [82]. The only gene previously found to influence behavior by modifying cue responsiveness is *Amfor* in honey bees, which regulates the onset of foraging by lowering the threshold for sucrose solution, a food-related stimulus [51,52]. In contrast to the *Amfor*-associated initiation of foraging, we provide evidence for early behavioral progression being regulated by an interplay between the pathways involving *Vg-like A*, worker age, and the relative responsiveness to specific chemical stimuli of brood and adult workers.

Based on gene expression patterns [34] and our finding that the expression of *Vg-like A* was linked to the initiation of brood care behavior, we conclude that, across all workers in a colony, brood carers exhibit the highest expression of *Vg-like A*. In unmanipulated colonies, typically the youngest workers take over brood care and are thus characterized by a high expression of *Vg-like A*, leading to a high sensitivity to brood stimuli and a high investment into brood care (Fig 1, [34]). After the hatching of a new worker generation in the summer, the recently hatched workers take over brood care duties and consequently replace the previous brood carers. The eclosion of new workers thus results in a temporary oversupply of brood carers and a reduction of the intensity of brood care–associated stimuli. Moreover, when young workers start emerging from the brood pile, previous brood carers are physically displaced from the center to the nest periphery, where the intensity of brood care stimuli is even lower [40]. As a consequence, the likelihood that previous brood carers are recruited to brood care tasks drops significantly once new workers start hatching [82,83]. Potentially facilitated via experience-based feedback loops [82–84] and at least partly influenced by a reduction in the expression of *Vg-like A*, chemical responsiveness is shifted from brood- to adult-worker cues. These former brood carers then direct their caring behavior toward adult nestmates, resulting in a behavioral repertoire including, e.g., intranidal hygenic behaviors and antennation of returning foragers at the nest entrance. This “push away” of workers from the brood pile is accompanied by a constant “pull out” of workers to outside tasks—e.g., by foragers dying in the field—and results in a behavioral progression from brood care to adult nestmate care and from adult nestmate care to foraging.

Such a push-pull model of behavioral progression has previously been proposed in honey bees [82]. We now expand this model by showing that the transition is internally influenced via *Vg-like A* and its associated pathways. These findings provide new evidence for proximate mechanisms underlying 2 prominent models of division of labor: *Vg-like A*–associated pathways increase responsiveness toward brood stimuli and can either explain the preferred area a worker is seeking a job in (FFW model [39]) or the preferred target of a certain behavior (RT [14,44]).

We present clear evidence that the progression from brood- to adult-nestmate care is at least partly controlled by a gene network including *Vg-like A*, whereas we do not know which genes control the subsequent steps of the behavioral progression. Foragers of the carpenter ant *Camponotus aethiops* behave more aggressively toward intruders than brood carers, which has been attributed to increased sensitivity toward nonnestmate stimuli [85]. In the ponerine ant *Harpegnathos saltator*, the neuropeptide corazonin has recently been shown to reduce *Vg* expression and to induce hunting behavior [86]. Whether corazonin takes over a similar role in *Temnothorax* ants remains unclear, as the RNA of this neuropeptide was not differentially expressed between brood carers and foragers in *T*. *longispinosus* [34]. Moreover, the *Vgs* of the two ponerine ants *H*. *saltator* and *Dinoponera quadriceps* form a monophyletic group, which is basal to all *Vg-like* genes, and they might therefore fulfill different functions.

After experimentally removing all recently hatched young workers, old workers compensated this loss by switching back to brood care. *Vg-like A* was more highly expressed in brood carers compared to foragers, independent of their age and fertility status [34]. Hence, a potential proximate mechanism underlying the behavioral reversal includes the up-regulation of *Vg-like A*, which was found in old reverted brood carers, compared to old workers who remained foragers (FDR = 0.0005). These reverted brood carers would then redirect their caring behavior from nestmates back to eggs, larvae, and pupae.

When removing all old workers from the nest, young workers failed to increase the frequency of nestmate care and foraging activity. This points to a constraint of young workers to accelerate their behavioral progression, e.g., via a down-regulation of *Vg-like A*. This is surprising because it cuts off the colony from resources. High thresholds for stimuli associated with tasks such as foraging might have prevented the young workers’ shift to outside tasks. Young workers have a higher life expectancy, fertility, and lipid reserves than old workers [14,15,17] and are thus more valuable for the colony. This is especially true for *Temnothorax* workers, which can live for several years [78] and thus have a high residual lifespan after hatching. To avoid the unnecessary exposure of valuable inside workers to elevated external mortality, selection should implement multiple thresholds that must be reached before workers switch to risky outside tasks. On a proximate level, young workers might only shift to foraging when they are pushed (by the emergence of new workers) and pulled by the shortage of resources. However, as young workers have large lipid reserves, they might respond to colony hunger slowly. It is therefore likely that old workers are more sensitive to cues, which cause them to switch back to inside tasks, than young workers to outside tasks.

Different orthologs of *CVg* and their pathways have extensively been studied, especially in honey bees, and are not only involved in fertility [87] but also in the regulation of brood care, the onset of foraging, longevity, immunocompetence, and the regulating of ageing [60–62,88,89]. Contrary to the studies on conventional *Vgs*, we provide first experimental evidence that *Vg-like A–*associated changes appear to be highly specialized in regulating responsiveness to worker and brood chemical cues. The knockdown of *Vg-like A* did not result in precocious foraging or forager-like CHC profiles, and we found no evidence for *Vg-like A* influencing fertility in workers or queens. The involvement of *Vg-like A* in immunocompetence and longevity (as has been suggested for honey bees [76]) still needs to be elucidated, although its expression was independent from worker age [34].

Moreover, our study confirms earlier ones indicating the existence of multiple copies of *Vg*s, including *Vg-likes* in ants [46,70–73]. *Vg* nomenclature has been rather inconsistent, with *Vg* genes and copies being consecutively numbered in successive genome annotations without clear structure. These inconsistencies hampered between-study comparisons of *Vg* genes, especially between *Vg* types of the “conventional” *Vgs* within ants. Our extensive BLAST search across all currently available ant genomes plus a number of additional social and solitary hymenopterans and other insects now resolves this and will help to clarify *Vg* classification in the future. Our phylogenetic tree provides the following novel insights: (a) *Vg-likes* (according to [73]) form 3 distinct clusters, with *Vg like A* and *B* being monophyletic, and they are present in all investigated insect species; (b) conventional *Vgs* form 2 distinct clusters with *CVg*, also being present in all investigated insect species, whereas the second cluster, *MVg*, is only found in ants—more specifically, only in myrmicine species. This finding suggests that *MVg* might have originated by duplication in an ancestor of the myrmicine ants but was consequently lost in some myrmicines. (c) The number of *Vgs* and *Vg-likes* varies strongly between ant, bee, and wasp taxa, which points to frequent gene duplications but also the recurrent loss of *Vg* genes. However, this finding could potentially be in part explained by incomplete genome assemblies. Although expression or functional data for most of the *Vg* and *Vg-like* genes are not available, the tissue-specific expression of these genes in *T*. *longispinosus* shown here and evidence from other ants [46,70,71,73] indicate different functions and caste-specific expression of the various *Vg* genes and may vary even between species.

In summary, we show how the expression of *Vg-like A* and its downstream network influences task choice of ant workers by modulating RTs to 2 social stimuli. We herewith discovered a mechanism that explains how age-dependent behavioral progression and behavioral flexibility can be achieved at the same time. The pathways involving *Vg-like A* might therefore have an important, but yet overlooked, impact on worker behavior and division of labor. *Vg-like A* is not only present in the genomes of social insects but also in those of solitary wasps and bees and in non-Hymenoptera taxa, including beetles and mosquitos. Hence, an across-taxa functional annotation of *Vg-like A* might shed more light on the evolution of brood care behavior in insects in general. Moreover, as the expression of *Vg-like A* in the fat body controlled the behavioral progression, our findings indicate that future research on the proximate basis of behavior should not only focus on the brain but also on other tissues and their cross communication.

## Material and methods

### Age polyethism and behavioral flexibility

In June 2014, 38 monogynous colonies of the ant *T*. *longispinosus* with an average colony size of 29.0 ± 1.5 workers were collected at the E. N. Huyck Preserve, Rensselearville, New York, United States of America, which also provided the collection permit. *T*. *longispinosus* colonies of this population have a synchronized annual brood production, with larval development taking about 1 year and new workers emerging July–August. At the time of collection, the brood of the previous year had not yet hatched; hence, all collected workers were at least 1 year old (termed “old” in the following). In our laboratories at the Johannes Gutenberg-University in Mainz, Germany, colonies were separately relocated into nests consisting of 2 glass slides separated by a piece of plexiglas providing a cavity of 4.9 cm × 1.1 cm × 0.3 cm. These slide nests were placed in 3-chambered boxes with a moistened plaster floor. Colonies were maintained at 14 h:10 h Light:Dark photoperiod and a +22 °C:+18 °C temperature regime to facilitate closing of the new worker generation. Ant colonies were fed twice a week with honey and pieces of crickets. In 24 randomly chosen colonies, all old workers were labeled with a thin metal wire (0.02 mm, Elektrisola) around the postpetiole. In the remaining 24 colonies, all young workers were labeled. Twenty-eight days after the hatching of the young workers was completed, colony demography was manipulated in 14 colonies per treatment group. Treatments included the removal of either (i) all freshly hatched workers in the lab (termed “young” in the following), (ii) all old workers, or (iii) half of each age cohort as a control. This manipulation aimed at forcing young workers to start foraging by the removal of the old foragers—and old workers to switch back to brood care—through the removal of young brood carers. Workers were then given 21 days to adapt to this manipulation and to reorganize their division of labor. To investigate the investment of single workers into brood care, nestmate care, and foraging, 6 foragers (observed being outside or in the nest entrance) and brood carers (observed actively conducting brood care when nest was opened) were individually marked with a thin colored metal. It has been shown in *T*. *longispinosus* and another ant species that a single observation is sufficient to group workers into brood carers and foragers differing in behavior [17,90], gene expression [34], CHC composition (S5 Fig), and life expectancy. Furthermore, spatial location has already been shown to predict behavior in *Temnothorax* ants [41]. Over the following 3 days, marked workers were scanned 10 times per day—i.e., a total of 30 times—by recording their position and behavior (S3 Table). We adhered to a period of at least 30 minutes between scans to increase independence of successive observations. Based on these data, relative brood care, nestmate care, and foraging activity was calculated for each worker. Brood care activity was defined as the “sum of antennating, grooming, feeding, or carrying of eggs, larvae, or pupae, divided by the total number of observations,” nestmate care activity as the “sum of antennating, grooming, feeding, or carrying of adult workers, divided by the total number of observations,” and foraging activity as the “number of times being observed outside the nest, divided by the total number of observations,” as ant workers mostly leave the nest to search for food. To test whether these three behaviors depended on age and/or demography treatment, 3 GLMMs were run, including relative “brood care activity,” “nestmate care activity,” or “foraging activity” as response variable and “age” (young, old), “demography treatment” (1 age cohort only, both age cohorts), and their interaction as explanatory factors. “Colony ID” was added as a random factor because multiple workers per colony were scanned. We used a quasi-Poisson distribution and log linkage function to correct for overdispersion.

### Identification of genes associated with behavior

A total of 48 full-body transcriptomes were collected from the “Age polyethism and behavioral flexibility” experiment. Here, we shortly summarize the methods and bioinformatic approaches; additional details are given in [34]. To identify genes exclusively associated with behavior, we sampled 24 brood carers and foragers each from both age cohorts and fertility levels. Samples were then sequenced on an Illumina HiSeq 2500, and raw reads were assembled using *CLC* Workbench (Qiagen) followed by a *MIRA* meta-assembly. We then ran a generalized linear model in *edgeR* v3.4 (Bioconductor) to detect differently expressed genes associated with “behavior” (brood carers versus forager), whereas age (young versus old) and fertility (fertile versus infertile) were added as blocking factors. cGMP-dependent protein kinase showed no caste-, age-, or fertility-dependent expression.

### *Vg-like A* regulation of behavior and behavioral progression

For these experiments, a total of 80 additional colonies were collected in June 2015 at the E. N. Huyck Preserve, NY, United States of America and kept under the previously described laboratory conditions. To assess phenotypic changes after RNAi-mediated *Vg-like A* knockdown, 2 brood carers, 2 inside workers (found inside the nest but not contributing to brood caring), 2 guards (found in the nest entrance), and 2 foragers were individually labeled with colored metal wire. In addition, 2 recently hatched workers were labeled to investigate age-dependent effects of RNAi-mediated gene knockdown. Ant colonies were moved to 25 °C and starved for 5 days to increase hunger, resulting in a more efficient uptake of dsiRNA. Open reading frame of the *Vg-like A* contig was extracted using NCBI *ORFfinder*, and three 25-bp-long dsiRNA fragments targeting different regions of the open reading frame (total contig length: 5511 bp; length of open reading frame: 4551 bp) were designed and synthesized by IDT, USA (S4 Table). Using multiple short fragments targeting the mRNA near the 5′ and 3′ end has been shown to increase knockdown efficiency [91,92]. As a control, we fed dsiRNA with no homologous region in the *T*. *longispinosus* transcriptome, though similar in length and nucleotide composition to the dsiRNA, which targets *Vg-like A*. For none of the 3 *Vg-like A* target sequences, we detected off-target binding in silico in the *T*. *longispinosus* transcriptome. These dsiRNA fragments also poorly align to all other *Vg* copies, except *Vg-like A*, which makes off-target effects unlikely (S5 Table). Fifteen μl of sucrose solution (0.102 g sucrose per 1 ml nuclease-free water) including 0.05 μg/μl per dsiRNA fragment were fed to the colony. As dsRNA remains stable in an ant’s crop for at least 24 hours [93], feeding solutions were renewed every day. Previously published protocols on administering dsRNA via feeding suggested to feed dsRNA in a concentration of 2 μg/μl. However, given the size differences between the large workers of *C*. *floridanus* (used in [93]) and the much smaller *T*. *longispinosus* (this study), the increased efficiency of the short dsiRNA compared to large dsRNA fragments, and to avoid overdosage, we decided to feed our dsiRNA in a concentration of 1 μg/μl. This concentration was additionally corrected for differences in fragment size ([93]: 400 bp. This study: 25 bp), i.e., divided by 20, resulting in a final concentration of 1/20 = 0.05 μg/μl. Per treatment (control, *Vg-like A* knockdown), 20 colonies were used. Nest scans were started after 3 days of feeding. Each colony was scanned 5 times per day for 4 days, i.e., a total of 20 scans. As we were unable to ensure that each worker received the same dosage of dsiRNA, we scanned several workers per colony and behavioral caste. During each scan, the position and behavior of each individually labeled ant was recorded (S3 Table). dsiRNA in sucrose solution was continuously fed during the observation days. To explore RNAi-induced changes in brood-caring behavior, a GLMM was run with “brood care frequency” (definition: see first paragraph of the Material and methods section) as response variable, “treatment” (*Vg-like A*^−^, control) and “caste” (brood carer, in-nest worker, guard, forager, young worker) as well as their interaction as explanatory variables, and “colony ID” as a random factor. The potential RNAi-mediated changes in “nestmate care,” “foraging frequency,” and “inactivity” (defined as the number of observations during which an individual was inactive) were tested with similar models.

### Individual brood care test

Feeding dsRNA to knock down a gene on a colony level can affect gene expression in larvae, suggesting that the dsRNA fragments are spread among colony members [93]. To test whether the observed changes in brood care could be explained by *Vg-like A* knockdown–induced alterations in larval behavior rather than by changes in worker behavior, a total of 65 brood care tests were conducted. Two brood carers per colony (a total of 36 colonies, half of them *Vg-like A*^−^, the other half control) were placed separately in a petri dish (diameter: 2 cm) containing a larva from a different colony, which was either part of the knockdown or control group before. The number and type of the interactions of the worker with the larva were recorded every 15 seconds for 5 minutes. This full factorial design allowed disentangling of the effect of worker and larval treatment on brood care behavior. For the statistical analysis, a GLMM was run including “brood care frequency” as response variable, “treatment of the worker” and “treatment of the larva” as well as their interaction as explanatory variable, and “colony ID of worker” and “larva” as random factors.

### Individual light/dark preference test

A total of 32 brood carers (16 control and 16 *Vg-like A*^−^ workers, all from different colonies) were transferred to a petri dish (diameter: 2 cm). Half of the petri dish was covered with black paint to create 2 distinct, equally sized darkened and lighted sections. The focal ant was placed in the center of the petri dish, and her position (i.e., which side of the petri dish) was recorded for 1 hour every 2 minutes resulting in a total of 30 observations per individual. A Welsh two-sample *t* test was used to compare the preference for the lighted side of the petri dish (number of observations that the individual was on this side) between control and *Vg-like A*^−^ workers.

### RNAi-induced long-term knockdown of *Vg-like A*

Twenty colonies per treatment (collected in 2015) were fed with *Vg-like A* dsiRNA or nonsense dsiRNA every second day for a total of 33 days. Nest scans following the protocol described in the “Age polyethism and behavioral flexibility” section were conducted between day 30 and day 33. To investigate effects of long-term knockdown of *Vg-like A*, a series of GLMMs were run, including either “frequency of brood care,” “foraging,” “inactivity,” or “nestmate care” as response variables; “treatment” (control, *Vg-like A* knockdown) as explanatory variable; and “colony ID” as a random factor. The application of dsiRNA over 33 days did not result in increased mortality of workers (Binomial GLM: χ^2^ = 1.8, *p* = 0.180), brood (Binomial GLM: χ^2^ = 0.3, *p* = 0.603), and queens (Binomial GLM: χ^2^ = 0.1, *p* = 0.747).

### Chemical profiles

After the last nest scan (day 33), 20 brood carers and foragers of control and *Vg-like A*^−^ colonies—i.e., a total of 80 workers—were individually frozen in glass vials for analysis of CHC profiles. CHCs were extracted and analyzed as described in [94]. Changes in the composition of the CHC profile were assessed using a PERMANOVA within the PRIMER software package (Quest Research).

### Fertility

After the long-term knockdown, all remaining queens and 1 individually labeled brood carer per colony were dissected, and length of all ovarioles was measured to the closest μm, using the Leica software, and the number of transparent and white eggs was counted. Apart from mean ovary length, we calculated “number of yolk-enriched eggs / (number of yolk-enriched eggs + number of transparent eggs)” to assess fertility. We used 2 GLMMs, including either “mean ovary length” or the “yolk-enriched egg / transparent egg” ratio as response variable, “treatment” (Control, *Vg-like A* knockdown) and “caste” (queen, worker) and their interaction as explanatory factors, and “colony ID” as a random factor.

### *Vg-like A*–associated responsiveness to task-related stimuli

In 29 colonies collected in July 2016 at the E. N. Huyck Preserve, NY, United States of America, 3 old (i.e., >1 year old) and 3 recently hatched young brood carers each were labeled with a thin metal wire, and *Vg-like A* was knocked down for 30 days, following the protocols described in the “*Vg-like A* regulation of behavior and behavioral progession” section. Old brood carers were identified by actively conducting brood care shortly before we started feeding dsiRNA. After that, 2 inside workers and 2 larvae were removed from the colony and freeze-killed for 24 hours at −20 °C, and CHCs were extracted in hexane for 10 minutes. These 4 extracts were then entirely transferred to 1 filter paper (diameter: 1 cm) each and provided as CHC samples in 2 subsequent behavioral tests per colony. For these tests, 1 old and 1 young brood carer per colony were individually transferred to a petri dish (diameter: 4 cm) and offered CHC samples of a worker and a larva from the same colony. For 10 minutes, interactions with both CHC samples were recorded. We then ran a binomial GLMM including the “number of interactions with each sample” as response; “age of the tested individual” (old, young), “treatment” (control, *Vg-like A* knockdown), and their interaction as explanatory variables; and “colony ID” as a random factor.

In each experiment, all behavioral data were collected by a single researcher. While recording the data, the observer was blind regarding (i) how the colony demography was manipulated, (ii) whether the colony was from the control or knockdown group, (iii) whether the individually labeled workers were young or old, and (iv) whether the individually labeled worker was classified as a brood carer or forager prior to the observation. All statistical analyses were (if not specifically mentioned) run in *R*, version 3.2.4, loaded with the packages *car*, *lme4*, and *MASS*, and GLMMs were modified using stepwise removal of nonsignificant interactions or main effects.

### Tissue-specific expression of *Vg* and *Vg-like* genes in *T*. *longispinosus*

Eight knockdown and control workers were individually homogenized in *Trizol* for qPCR analysis. RNA was extracted using RNeasy Mini Kit (Qiagen), and cDNA was synthesized with QuantiTect Rev. Transcription Kit (Qiagen). We then amplified *Vg-like A* as well as *alpha tubulin* (*AT*) and *GAPDH* as housekeeping genes (HKGs, S6 Table) to correct for different quantities of input material using SensiFAST SYBR Hi-ROX Kit (Bioline). We used the RNAseq dataset that revealed *Vg-like A* as an interesting candidate gene [34] to show that the expression of *AT* and *GAPDH* is independent from whether a worker serves as a brood carer or forager (*AT*: FDR = 0.333; *GAPDH*: FDR = 0.907), whether the worker is young or old (*AT*: FDR = 0.830; *GAPDH*: FDR = 0.578), and whether the worker is fertile or infertile (*AT*: FDR = 0.999; *GAPDH*: FDR = 0.999). Moreover, the expression of *Vg-like A* was unlinked to the expression of *AT* (Pearson: *p* = 0.228) and *GAPDH* (Pearson: *p* = 0.259), suggesting that a knockdown of *Vg-like A* does not result in expression changes in either of the 2 HKGs. The expression of *AT* and *GAPDH* was tightly correlated (Pearson: *p* = 0.0004). To further test whether *AT* and *GAPDH* are expressed similarly among brood carers and foragers, we used a total of 8 colonies of *T*. *longispinosus*, extracted RNA of 1 brood carer and 1 forager per colony, and constructed cDNA libraries. We quantified DNA content of each cDNA library and diluted each library to 0.0002 μg/μl and quantified *AT* and *GAPDH* using 10 μl of each diluted library, using qPCR. We ran 2 GLMMs to test whether the expression of *AT* or *GAPDH* depends on “behavioral caste” (brood carer versus forager), using “colony ID” as a random factor: The expression of *AT* (GLMM: *p* = 0.847) and *GAPDH* (*p* = 0.861) was not different between both castes. Moreover, we used Bestkeeper [95] to show that both genes exhibit stable expression patterns (*AT*: SD = 0.45; *GAPDH*: SD = 0.4). We additionally analyzed our tissue-specific qPCR data using Bestkeeper to test whether both HKGs exhibit stable expression across tissues. *AT* was equally expressed in all tissues (SD = 0.91), and *GAPDH* showed a weak tissue-specific expression (SD = 1.04). qPCR results were then analyzed using ΔΔCT method and Wilcoxon test to compare relative expression of the targeted gene between treatment and control workers. *Vg-like A*–specific primers amplify a fragment near the spliced-off 3′ end, which increases the likelihood to detect an RNAi-induced down-regulation [92].

To assess tissue-specific expression, we labeled 5 brood carers in a total of 22 colonies. In 11 colonies, we knocked down *Vg-like A* for 7 days, following the protocol described in the “*Vg-like A* regulation of behavior and behavioral progession” section. The remaining 11 colonies were fed with nonsense dsiRNA. Five brood carers per colony were dissected, and ovaries, fat body, and brains, respectively, were pooled in Trizol to minimize the effect of individual variation. Samples were then processed and analyzed as described in the “Tissue-specific expression of *Vg* and *Vg-like* genes in *T*. *longispinosus”* section. To further investigate potential effects of *Vg-like A* dsiRNA application on the expression of other *Vg* copies, we also quantified *VgC*, *MVg*2, *MVg*3, *Vg*-*like B*, and *Vg*-*like C*, using qPCR (S6 Table). Tissue-specific qPCR data were analyzed using the ΔΔCT method followed by 2 GLMMs per gene (1 for each HKG), including relative expression as a response variable; “tissue” (brain, fat body, ovaries), “treatment” (control, *Vg-like A* knockdown), and their interaction as explanatory factors; and “colony ID” as a random factor. To further assess tissue specificity in the expression of each gene, we ran 1 GLMM per gene on the control colonies, including relative expression of the respective gene as a response and “tissue” as an explanatory factor. “Colony ID” was added as a random factor. As there were no qualitative differences between both HKGs, only results for *AT* were shown (results for *GAPDH* are summarized in S6 Fig).

### Resolving the *Vg* and *Vg-like* phylogeny in ants and hymenopterans

*Vg* orthologs have previously been grouped into *Vgq* (associated with reproduction) and *Vgw* (associated with brood care) [72] or *Vg-like* and conventional *Vgs* [52]. Our BLAST searches of the *T*. *longispinosus Vg* contigs were highly ambiguous. We therefore conducted BLAST searches of the *Vg* sequences published by Oxley and colleagues [50] and Morandin and colleagues [52] to identify *Vg* genes in all ant species with a currently available genome plus a number of social and nonsocial hymenopterans and other insects (S1 Table) and *T*. *longispinosus* contigs [34]. In several species, we detected “fused” *Vg* protein sequences—i.e., the genome annotation identified a single *Vg* gene—although it actually contained 2 open reading frames. We split these fused proteins in their 2 according subparts. All previously published *Vg* sequences plus *Vg* sequences obtained by BLAST searches were used to construct an alignment using the web-based Clustal Omega tool (https://www.ebi.ac.uk/Tools/msa/clustalo/). A maximum-likelihood tree was constructed with RAxML (8.2.11 [75]), using the PROTGAMMAAUTO to automatically identify the best-fitting protein model (JTT), and 1,000 bootstraps. For additional versions of the phylogenetic tree, including full species names and NCBI reference sequence ID system labels, see S7 and S8 Figs.

## Supporting information

S1 Fig “Frequency of inactivity” did not differ between control and *Vg-like A*^−^ workers during short-term knockdown experiment. Inactivity was measured as the number of observations during which an individual was not showing any behavior. We then ran a GLMM including “frequency of inactivity” as a response variable; “treatment” (control, *Vg-like A*^−^), “caste,” and their interaction as explanatory factors; and “colony ID” as a random factor. The interaction of “treatment” and “caste,” as well as “treatment” as a main factor, had no effect on inactivity (interaction, GLMM: χ^2^ = 5.5, *p* = 0.24; “treatment,” GLMM: χ^2^ = 1.3, *p* = 0.255). Caste-dependent differences in inactivity were found (GLMM: χ^2^ = 24, *p* < 0.0001), which we did not explore in more detail. Orange: control. Purple: *Vg-like A* knockdown. GLMM, generalized linear mixed model; *Vg*, vitellogenin.
(PDF)

S2 Fig “Frequency of foraging” was not influenced by short-term *Vg-like A* knockdown. Orange: control. Purple: *Vg-like A* knockdown. *Vg*, vitellogenin.
(PDF)

S3 Fig Brood care in young brood carers was strongly reduced after 33 days of *Vg-like A* knockdown (GLMM: *p* < 0.0001). Orange: control. Purple: *Vg-like A* knockdown. GLMM, generalized linear mixed model; *Vg*, vitellogenin.
(PDF)

S4 Fig Foraging frequency in young brood carers did not change in response to 33 days of *Vg-like A* knockdown (GLMM: *p* = 0.79). Orange: control. Purple: *Vg-like A* knockdown. GLMM, generalized linear mixed model; *Vg*, vitellogenin.
(PDF)

S5 Fig NMDS plots of CHC profiles of brood carers and foragers and *Vg-like A* knockdown and control workers. (A) NMDS plot of the composition of CHC profile differed between brood carers (blue) and foragers (red). (B) A knockdown of *Vg-like A* (purple) did not result into alterations of CHC profiles compared to control workers (orange). CHC, cuticular hydrocarbon; NMDS, nonmetric dimensional scaling; *Vg*, vitellogenin.
(PDF)

S6 Fig Expression relative to *GAPDH* of all *Vg* and *Vg-like* genes found in *Temnothorax*. Orange: control. Purple: *Vg-like A* knockdown. *Vg*, vitellogenin.
(TIF)

S7 Fig Phylogenetic tree of *Vg* and *Vg-like* orthologs with full species names. *Vg*, vitellogenin.
(TIF)

S8 Fig Phylogenetic tree of *Vg* and *Vg-like* with NCBI reference sequence ID system labels. *Vg*, vitellogenin.
(SVG)

S1 Table Overview of different *Vg* and *Vg-like* copies per species. Numbers represent the numbers of gene copies per cluster found. *Vg*, vitellogenin.
(PDF)

S2 Table Overview of fused protein sequences detected in previously published genome annotations. (PDF)

S3 Table Overview of the behaviors and positions recorded during nest scans. Brood caring was defined as the number of observations during which a worker antennated, groomed, fed, or carried a brood item. Nestmate care was the sum of antennating, grooming, feeding, or carrying adult nestmate workers. Foraging was defined as the number of observations during which an individual was found outside the nest.
(PDF)

S4 Table dsiRNA sequences. Frequency of nucleotides: *Vg-like A* (median across all 3 fragments): A = 5; C = 6; G = 6; U = 8. Nonsense: A = 5; C = 6; G = 6; U = 8. dsiRNA; Dicer-substrate small interfering RNA; *Vg*, vitellogenin.
(PDF)

S5 Table Specificity of *Vg-like A* dsiRNA fragments. Alignment was performed using EMBL-EBIs *Clustal Omega*. *Vg*, vitellogenin.
(PDF)

S6 Table qPCR primer sequences. qPCR, quantitative real-time PCR.
(PDF)

## Abbreviations

*AT*: *alpha tubulin*
CHC: cuticular hydrocarbon
dsiRNA: Dicer-substrate small interfering RNA
FDR: false discovery rate
FFW: foraging for work
GLMM: generalized linear mixed model
HKG: housekeeping gene
IGF-1: insulin-like growth factor 1
JH: juvenile hormone
PERMANOVA: permutational multivariate ANOVA
qPCR: quantitative real-time PCR
RNAi: RNA interference
RT: response threshold
*Vg*: *vitellogenin*
