# Supplementary figures and images for: Vitellogenin-like A–associated shifts in social cue responsiveness regulate behavioral task specialization in an ant
Source: PLoS Biol. 2018 Jun 6;16(6):e2005747. doi: 10.1371/journal.pbio.2005747 (PMC5991380; doi:10.1371/journal.pbio.2005747)

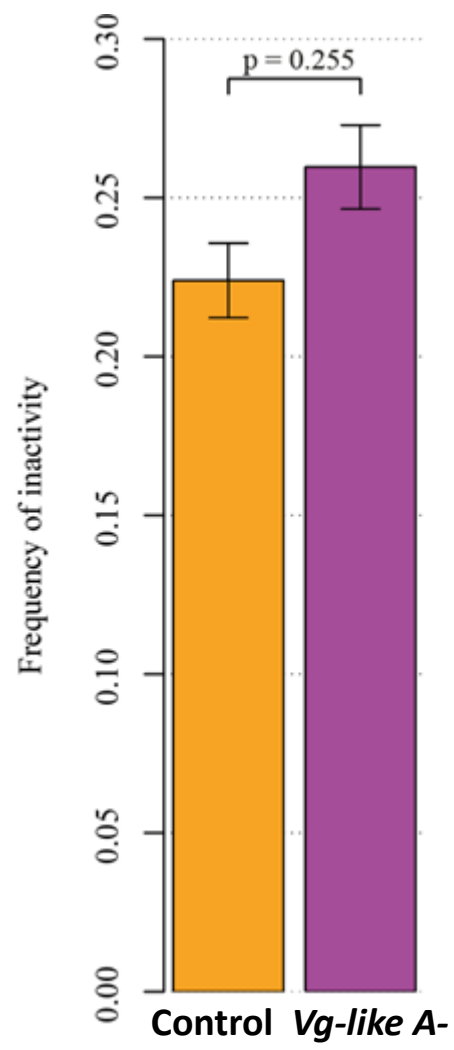

Supplement: S1 Fig — Inactivity was measured as the number of observations during which an individual was not showing any behavior. We then ran a GLMM including “frequency of inactivity” as a response variable; “treatment” (control, Vg-like A−), “caste,” and their interaction as explanatory factors; and “colony ID” as a random factor. The interaction of “treatment” and “caste,” as well as “treatment” as a main factor, had no effect on inactivity (interaction, GLMM: χ2 = 5.5, p = 0.24; “treatment,” GLMM: χ2 = 1.3, p = 0.255). Caste-dependent differences in inactivity were found (GLMM: χ2 = 24, p < 0.0001), which we did not explore in more detail. Orange: control. Purple: Vg-like A knockdown. GLMM, generalized linear mixed model; Vg, vitellogenin. (PDF) [file pbio.2005747.s001.pdf]

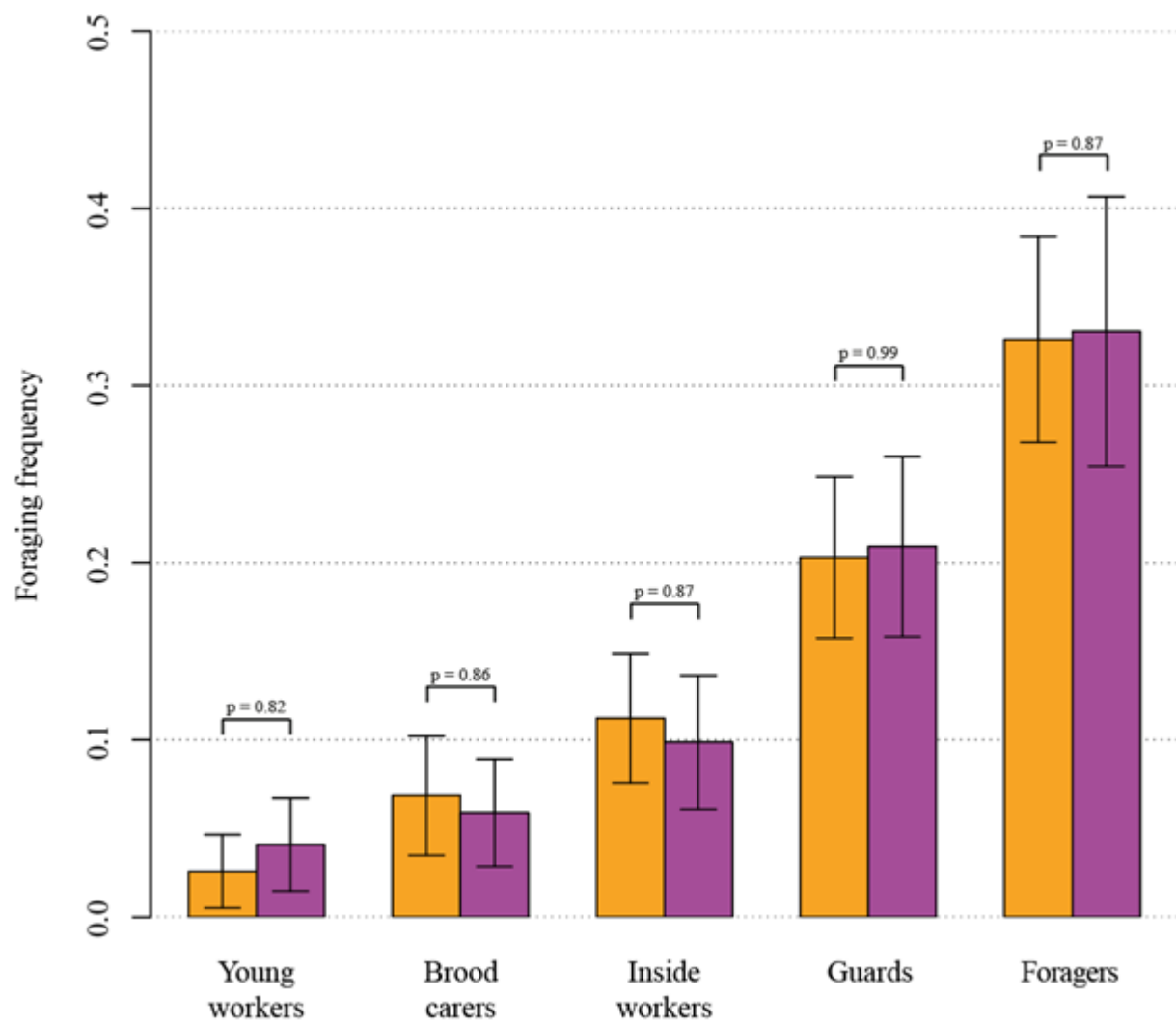

Supplement: S2 Fig — Orange: control. Purple: Vg-like A knockdown. Vg, vitellogenin. (PDF) [file pbio.2005747.s002.pdf]

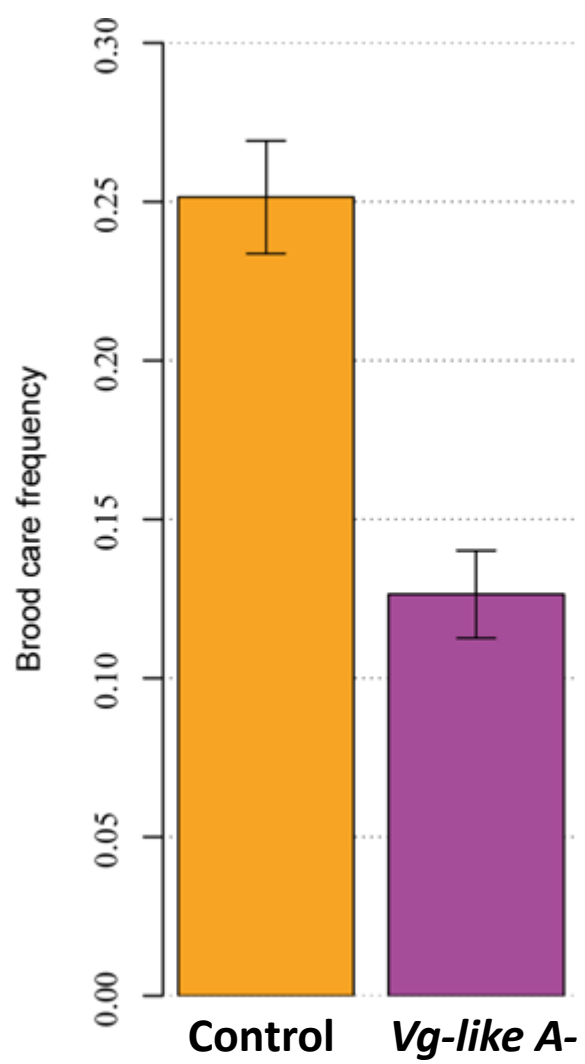

Supplement: S3 Fig — Orange: control. Purple: Vg-like A knockdown. GLMM, generalized linear mixed model; Vg, vitellogenin. (PDF) [file pbio.2005747.s003.pdf]

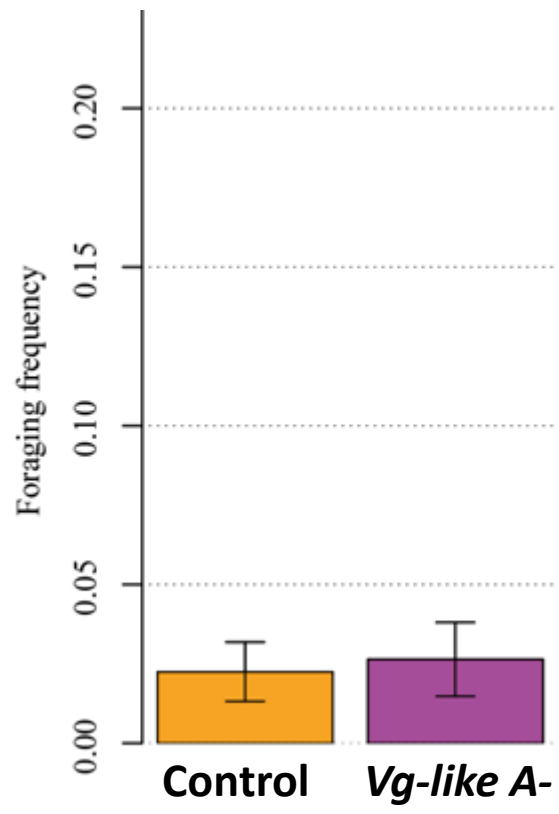

Supplement: S4 Fig — Orange: control. Purple: Vg-like A knockdown. GLMM, generalized linear mixed model; Vg, vitellogenin. (PDF) [file pbio.2005747.s004.pdf]

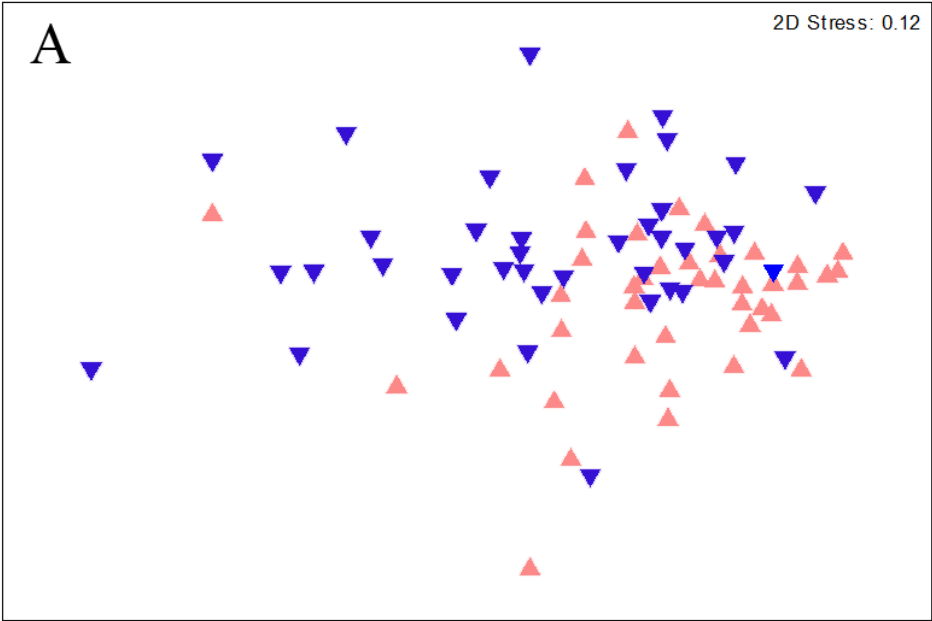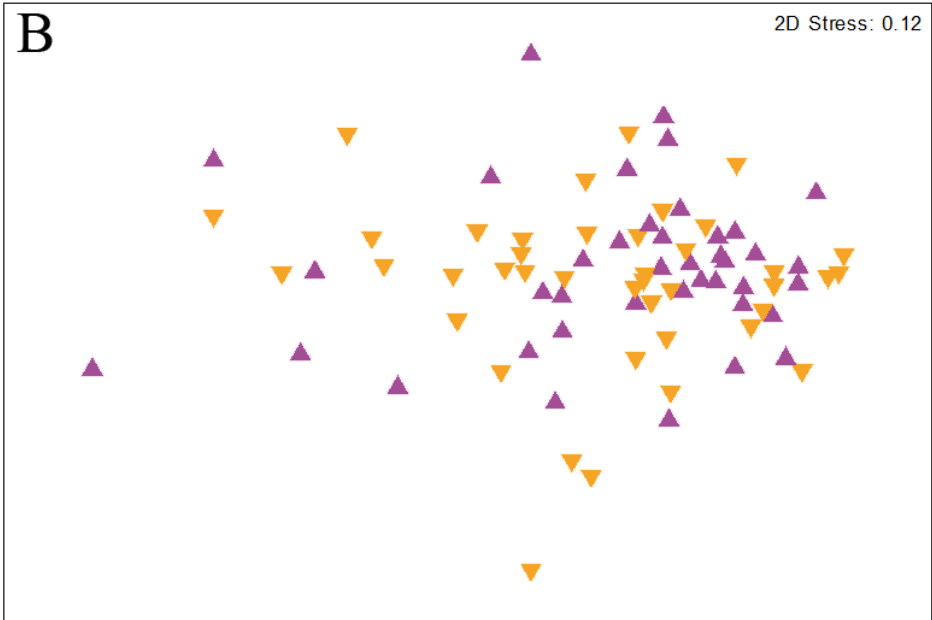

Supplement: S5 Fig — (A) NMDS plot of the composition of CHC profile differed between brood carers (blue) and foragers (red). (B) A knockdown of Vg-like A (purple) did not result into alterations of CHC profiles compared to control workers (orange). CHC, cuticular hydrocarbon; NMDS, nonmetric dimensional scaling; Vg, vitellogenin. (PDF) [file pbio.2005747.s005.pdf]

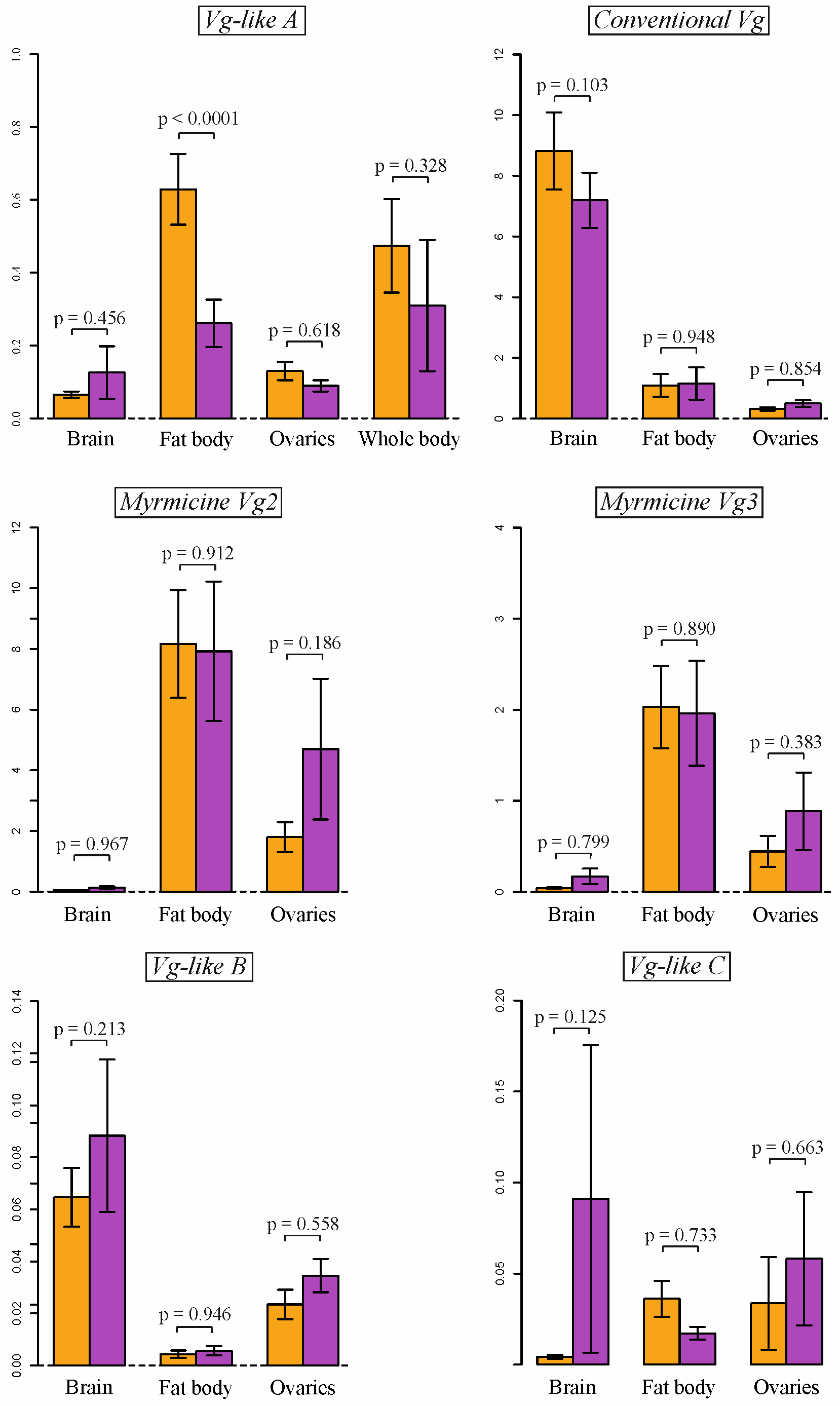

Supplement: S6 Fig — Orange: control. Purple: Vg-like A knockdown. Vg, vitellogenin. (TIF) [file pbio.2005747.s006.tif]

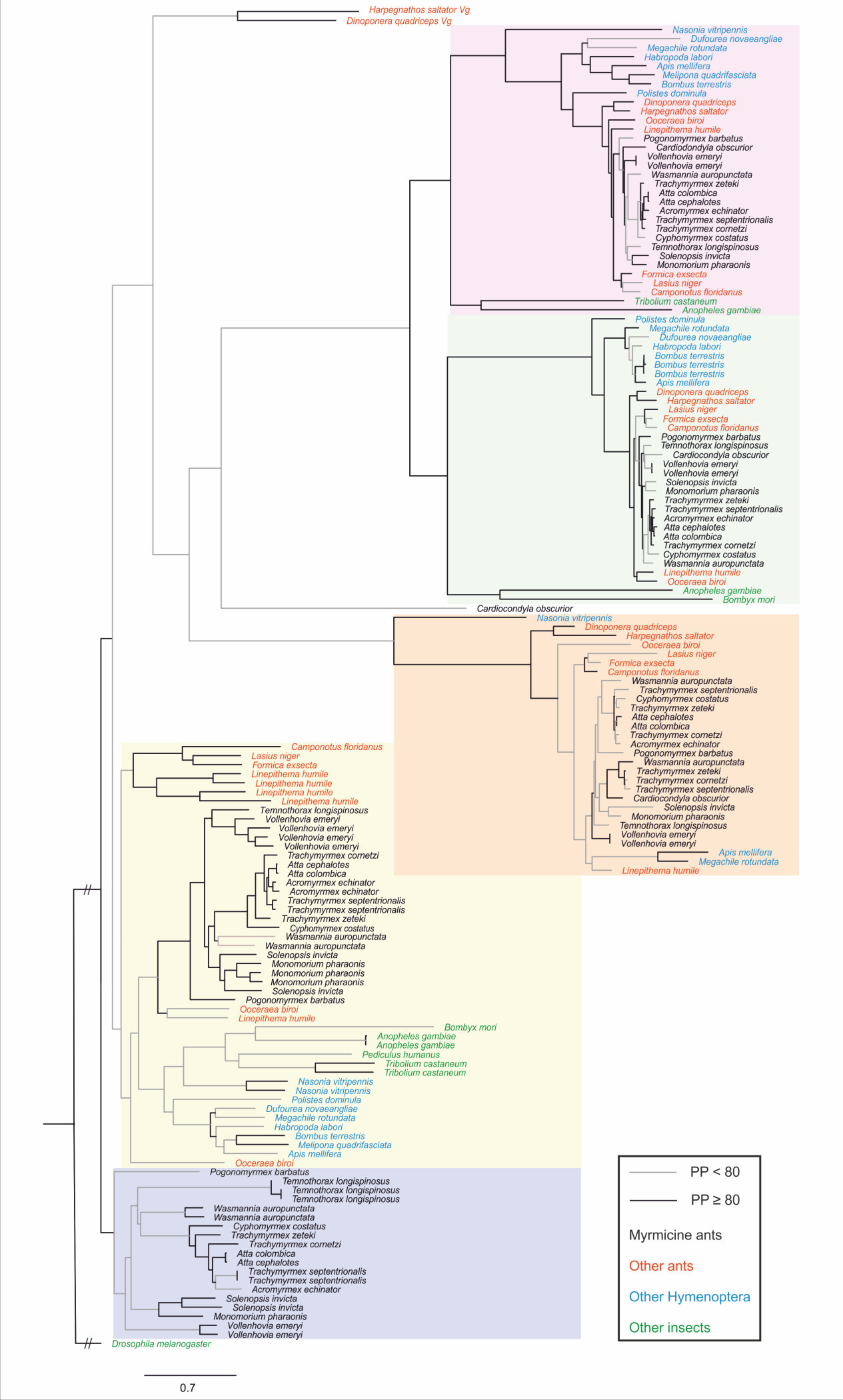

Supplement: S7 Fig — Vg, vitellogenin. (TIF) [file pbio.2005747.s007.tif]
