# Supplementary material for: Vitellogenin-like A–associated shifts in social cue responsiveness regulate behavioral task specialization in an ant
Source: PLoS Biol. 2018 Jun 6;16(6):e2005747. doi: 10.1371/journal.pbio.2005747 (PMC5991380; doi:10.1371/journal.pbio.2005747)
Supplement: S1 Table — Numbers represent the numbers of gene copies per cluster found. Vg, vitellogenin. (PDF) [file pbio.2005747.s009.pdf]

**Species**	***Conventional Vg***	***Vg-like A***	***Vg-like B***	***Vg-like C***	***Myrmicine Vg***	***Unclustered Vg***
Acromyrmex echinatior	**2**	**1**	**1**	**1**	**2**	**0**
Anopheles gambiae	**2**	**1**	**1**	**0**	**0**	**0**
Atta cephalotes	**1**	**1**	**1**	**1**	**1**	**0**
Atta colombica	**1**	**1**	**1**	**1**	**0**	**0**
Apis mellifera	**1**	**1**	**1**	**1**	**0**	**0**
Bombus terrestris	**1**	**1**	**3**	**0**	**0**	**0**
Bombyx mori	**1**	**0**	**1**	**0**	**0**	**0**
Camponotus floridanus	**1**	**1**	**1**	**1**	**0**	**0**
Cardiocondyla obscurior	**0**	**1**	**1**	**1**	**0**	**1**
Ooceraea biroi	**2**	**1**	**1**	**1**	**0**	**0**
Cyphomyrmex costatus	**1**	**1**	**1**	**1**	**1**	**0**
Dinoponera quadriceps	**0**	**1**	**1**	**1**	**0**	**1**
Drosophila melanogaster	**0**	**0**	**0**	**0**	**0**	**1**
Dufourea novaeangliae	**1**	**1**	**1**	**0**	**0**	**0**
Formica exsecta	**1**	**1**	**1**	**1**	**0**	**0**
Habropoda labori	**1**	**1**	**1**	**0**	**0**	**0**
Harpegnathos saltator	**0**	**1**	**1**	**1**	**0**	**1**
Lasius niger	**1**	**1**	**1**	**1**	**0**	**0**

Linepithema humile	**5**	**1**	**1**	**1**	**0**	**0**
Megachile rotundata	**1**	**1**	**1**	**1**	**0**	**0**
Melipona quadrifasciata	**1**	**1**	**0**	**0**	**0**	**0**
Monomorium pharaonis	**3**	**1**	**1**	**1**	**1**	**0**
Nasonia vitripennis	**2**	**1**	**0**	**1**	**0**	**0**
Pediculus humanus	**1**	**0**	**0**	**0**	**0**	**0**
Pogonomyrmex barbatus	**1**	**1**	**1**	**1**	**1**	**0**
Polistes dominula	**1**	**1**	**1**	**0**	**0**	**0**
Solenopsis invicta	**2**	**1**	**1**	**1**	**2**	**0**
Temnothorax longispinosus	**1**	**1**	**1**	**1**	**2**	**0**
Trachymyrmex cornetzi	**1**	**1**	**1**	**2**	**1**	**0**
Trachymyrmex septentrionalis	**2**	**1**	**1**	**2**	**2**	**0**
Trachymyrmex zeteki	**1**	**1**	**1**	**2**	**1**	**0**
Tribolium castaneum	**2**	**1**	**0**	**0**	**0**	**0**
Vollenhovia emeryi	**4**	**2**	**2**	**2**	**2**	**0**
Wasmannia auropunctata	**2**	**1**	**1**	**2**	**2**	**0**
