# Supplementary material for: Vitellogenin-like A–associated shifts in social cue responsiveness regulate behavioral task specialization in an ant
Source: PLoS Biol. 2018 Jun 6;16(6):e2005747. doi: 10.1371/journal.pbio.2005747 (PMC5991380; doi:10.1371/journal.pbio.2005747)
Supplement: S2 Table — (PDF) [file pbio.2005747.s010.pdf]

Species	Sequence ID	Annotation of first ORF	Cluster of first ORF	Annotation of second ORF	Cluster of second ORF
P.barbatus	>Pbar_Yp1-1	*Vg1*	*VgM*	*Vg2*	*VgC*
T. cornetzi	>XP_018374343.1	*Vg2*	*VgM*	*Vg1*	*VgC*
V. emeryi	>XP_011879089.1	*Vg1*	*VgM*	*Vg3*	*VgC*
