# Supplementary material for: Vitellogenin-like A–associated shifts in social cue responsiveness regulate behavioral task specialization in an ant
Source: PLoS Biol. 2018 Jun 6;16(6):e2005747. doi: 10.1371/journal.pbio.2005747 (PMC5991380; doi:10.1371/journal.pbio.2005747)
Supplement: S3 Table — Brood caring was defined as the number of observations during which a worker antennated, groomed, fed, or carried a brood item. Nestmate care was the sum of antennating, grooming, feeding, or carrying adult nestmate workers. Foraging was defined as the number of observations during which an individual was found outside the nest. (PDF) [file pbio.2005747.s011.pdf]

**Behaviors**	**Positions**
Resting	On the brood pile
Walking	Near the brood pile (One body length)
Grooming (Self, brood, adult worker queen)	Inside the nest
Feeding (Brood, adult worker, queen)	Near the entrance (One body length)
Carrying (Brood, adult worker queen)	In the nest entrance
Antennation (Nest, brood, adult worker, queen)	Outside chamber 1
Being fed	Outside chamber 2
Being groomed	Outside chamber 3
Eating	
