# Supplementary material for: Vitellogenin-like A–associated shifts in social cue responsiveness regulate behavioral task specialization in an ant
Source: PLoS Biol. 2018 Jun 6;16(6):e2005747. doi: 10.1371/journal.pbio.2005747 (PMC5991380; doi:10.1371/journal.pbio.2005747)
Supplement: S4 Table — Frequency of nucleotides: Vg-like A (median across all 3 fragments): A = 5; C = 6; G = 6; U = 8. Nonsense: A = 5; C = 6; G = 6; U = 8. dsiRNA; Dicer-substrate small interfering RNA; Vg, vitellogenin. (PDF) [file pbio.2005747.s012.pdf]

**Fragment**	**Direction**	**Sequence**
Vg-like A fragment 1	Sense	CCAUCAACAGGCUACAGCAUACUAU
	Antisense	AUAGUAUGCUGUAGCCUGUUGAUGGUU
Vg-like A fragment 2	Sense	CCAUGAAUUGCGUCCGUGAGACUUU
	Antisense	AAAGUCUCACGGACGCAAUUCAUGGUU
Vg-like A fragment 3	Sense	CGAGUACGUUCAAGUUCCUUUGCAG
	Antisense	CUGCAAAGGAACUUGAACGUACUCGUA
Nonsense	Sense	UAGUACACGUCAUUGGAAUUGCAGC
	Antisense	GCUGCAAUUCCAAUGACGUGUACUAUU
