# Supplementary material for: Vitellogenin-like A–associated shifts in social cue responsiveness regulate behavioral task specialization in an ant
Source: PLoS Biol. 2018 Jun 6;16(6):e2005747. doi: 10.1371/journal.pbio.2005747 (PMC5991380; doi:10.1371/journal.pbio.2005747)
Supplement: S5 Table — Alignment was performed using EMBL-EBIs Clustal Omega. Vg, vitellogenin. (PDF) [file pbio.2005747.s013.pdf]

Gene	*Vg-like A* dsiRNA fragment	Proportion of correctly aligned nucleotides	Alignment of the longest aligned fragment
*Vg*1	1	68%	accatgaacacacaacagctgactgaa<br>-CCATCAACAGGCTACAGCATACTAT-<br>**** **** * ***** ***
*Vg*1	2	64%	cgcaagaattaaatccgcgaggtgttc<br>-CCATGAATTGCGTCCGTGAGACTTT-<br>** ***** **** *** **
*Vg*1	3	60%	gagtgtcagtcgaggtcccttagcggc<br>-CGAGTACGTTCAAGTTCCTTTGCAG-<br>* ** ** * ** **** ** *
*Vg*2	1	48%	CGTCCCGATGGTAACTCATAGCATT<br>-ATCAACAGGCTACAGCATACTAT-<br>** * * ** **** **
*Vg*2	2	52%	TCATAGCATTGGGCTTGTTCAGCTTTC<br>-CCATGAATTGCGTCCGTGAGACTTT-<br>* * **** * ** ****
*Vg*2	3	60%	TCGACTGTCTTCCTGCTTCTTTGTCGC<br>-CGAGTACGTTCAAGTTCCTTTGCAG-<br>*** * *** * * ***** *
*Vg*3	1	28%	GGAACCACATGGTGTT<br>-CTACAGCATACTAT-<br>** *** * *
*Vg*3	2	60%	gtaatgtattgtgtatatgagaatact<br>-CCATGAATTGCGTCCGTGAGACTTT-<br>*** **** ** ***** *
*Vg*3	3	32%	atgtcccttttccc<br>-AGTTCCTTTGCA-<br>** ***** *
Vg-like A	1	100%	ACCATCAACAGGCTACAGCATACTATG<br>-CCATCAACAGGCTACAGCATACTAT-<br>*************************
Vg-like A	2	100%	ACCATGAATTGCGTCCGTGAGACTTTA<br>-CCATGAATTGCGTCCGTGAGACTTT-<br>*************************
Vg-like A	3	100%	ACGAGTACGTTCAAGTTCCTTTGCAGC<br>-CGAGTACGTTCAAGTTCCTTTGCAG-<br>*************************
Vg-like B	1	68%	gcgttctatagacttcagcatcgtata<br>-CCATCAACAGGCTACAGCATACTAT-<br>* ** * ** ** ****** ***
Vg-like B	2	64%	accaggattttcgagcctgtggcttaa<br>-CCATGAATTGCGTCCGTGAGACTTT-<br>*** ** ** ** * ** * ***
Vg-like B	*3*	*44%*	gcgagttcattagactt<br>-CGAGTACGTTCAAGTT<br>***** * ** * **
Vg-like C	*1*	*44%*	gcctacattatattatc[illegible]<br>-GCTACAGCATACTAT-[illegible]<br>***** *** ***

Vg-like C	*2*	*44%*	atcacgtacgttataattatc<br>-TTGCGTCCGTGAGACTTT--<br>* *** *** * * **
Vg-like C	*3*	*36%*	caaattcgtgggcagtg<br>--AGTTCCTTTGCAG--<br>* *** * ****
