# Supplementary material for: Vitellogenin-like A–associated shifts in social cue responsiveness regulate behavioral task specialization in an ant
Source: PLoS Biol. 2018 Jun 6;16(6):e2005747. doi: 10.1371/journal.pbio.2005747 (PMC5991380; doi:10.1371/journal.pbio.2005747)
Supplement: S6 Table — qPCR, quantitative real-time PCR. (PDF) [file pbio.2005747.s014.pdf]

Gene	Direction	Sequence
Vg-like A	5’ to 3’	TGGAGATCATGCCAAACGAG
	3’ to 5’	CGTTGAGACTGGACCATGAG
Conventional Vg	5’ to 3’	TCTCTTCAAGACGCCAGAATTAG
	3’ to 5’	CCGACGATACCAGAACGATAAG
VgM (Vg2)	5’ to 3’	GTTGATGGCAGACCAGACGA
	3’ to 5’	TGAAACAGAGCTCGTCACCC
VgM (Vg3)	5’ to 3’	ACTTTGGCACTCGGAGGAAG
	3’ to 5’	TTGTGCAAGCTTCTCAACGC
Vg-like B	5’ to 3’	TGCTCGAGACGCCTCAATTT
	3’ to 5’	TGCGTATTCCACAGGGCATT
Vg-like C	5’ to 3’	TTCTCGCAATCGTCCTTGCT
	3’ to 5’	ACTCCTCCAGACTCCTCGAC
Alpha tubulin	5’ to 3’	TCCATCGAACCGTAGAGAGG
	3’ to 5’	GCAACCTGGATATCGAAAGG
GDAPH	5’ to 3’	TCATCTTCGGTGTAGCCAG
	3’ to 5’	CGACTTGACGGTTCGACTTG
